# In vitro and in vivo assessment of caprine origin Staphylococcus aureus ST398 strain UTCVM1 as an osteomyelitis pathogen

**DOI:** 10.3389/fcimb.2022.1015655

**Published:** 2022-11-24

**Authors:** Caroline Billings, Rebecca Rifkin, Mohamed Abouelkhair, Rebekah Duckett Jones, Austin Bow, Jaydeep Kolape, Sreekumari Rajeev, Stephen Kania, David E. Anderson

**Affiliations:** ^1^ Large Animal Clinical Sciences, College of Veterinary Medicine, University of Tennessee, Knoxville, TN, United States; ^2^ Biomedical and Diagnostic Sciences, College of Veterinary Medicine, University of Tennessee, Knoxville, TN, United States; ^3^ Advanced Microscopy and Imaging Center, University of Tennessee, Knoxville, TN, United States

**Keywords:** Osteomyelitis, *Staphylococcus aureus*, *in vitro*, *in vivo*, intracellular invasion

## Abstract

*Staphylococcus aureus* (SA) is a significant and well-recognized causative organism of bacterial osteomyelitis. Osteomyelitis is an inflammatory bone disease characterized by progressive bone destruction and loss. This disease causes significant morbidity and mortality to the patient and poses therapeutic challenges for clinicians. To improve the efficacy of therapeutic strategies to combat bacterial osteomyelitis, there is a need to define the molecular epidemiology of bacterial organisms more clearly and further the understanding of the pathogenesis of SA osteomyelitis. We conducted *in vitro* characterization of the pathogenic capabilities of an isolate of SA ST398 derived from a clinical case of osteomyelitis in a goat. We also report a rodent mandibular defect model to determine the ability of ST398 to cause reproducible osteomyelitis. Our results indicate that ST398 can invade and distort pre-osteoblastic cells in culture, induce significant inflammation and alter expression of osteoregulatory cytokines. We also demonstrate the ability of ST398 to induce osteomyelitis in a rat mandibular model. When compiled, these data support ST398 as a competent osteomyelitis pathogen.

## 1 Introduction

Osteomyelitis is an inflammatory bone disease, typically caused by bacteria, that causes progressive bone destruction and loss (Kavanagh et al., 2018). This destructive process results in significant morbidity and mortality to affected individuals (Lew and Waldvogel, 2004). *Staphylococcus aureus* (SA), a common commensal organism (Kluytmans et al., 1997; Tong et al., 2015), is also a versatile and dangerous human pathogen (Lowy, 1998; Tong et al., 2015), and is responsible for the majority of bacterial osteomyelitis cases (Lew and Waldvogel, 2004; Wright and Nair, 2010; Kavanagh et al., 2018; Pimentel de Araujo et al., 2021). Various etiologies of osteomyelitis are recognized, including 1. hematogenous, 2. secondary to trauma or invasive procedures such as surgery and implantation of indwelling medical implants, and 3. secondary to vascular insufficiency, such as diabetic foot ulcers (Fritz and McDonald, 2008; Kluin et al., 2013). Regardless of etiology, osteomyelitis is a serious condition that warrants swift and intensive therapy. Therapeutic strategies for acute and chronic osteomyelitis are heavily reliant upon systemic antimicrobial therapy which may be complemented by local antimicrobial therapy (Kluin et al., 2013; Urish and Cassat, 2020). Standards of practice for treatment of osteomyelitis, especially in chronic cases, also include surgical debridement of affected and devitalized tissues, which may involve removal of implanted hardware (Lew and Waldvogel, 2004). Despite rapid and appropriate intervention (Hogan et al., 2013), osteomyelitis often becomes chronic, a phenomenon due in large part to the pathogenic capabilities of SA (Masters et al., 2019).

The ability of SA to create chronic, recalcitrant, or recurrent osteomyelitis (Libraty et al., 2012) through virulence factors and immune system evasion is multifactorial (Tucker et al., 2000; Al-Maiyah et al., 2007; Liu, 2009; Post et al., 2014; Masters et al., 2019; Muthukrishnan et al., 2019) and incompletely understood. Mechanisms of immune system evasion include Staphylococcal abscess formation, biofilm formation, osteocyte-lacuno canalicular network (OLCN) invasion, as well as intracellular invasion and persistence (Masters et al., 2019; Muthukrishnan et al., 2019; Masters et al., 2021). There is a need to gain deeper understanding of immune system evasion, particularly the significance of intracellular invasion. Recently, it has been reported that the ability of SA to gain intracellular osteoblast access may not only contribute to immune system evasion but may also play a role in shifting osteoblastic activity to induce inflammatory bone pathology (Alexander and Hudson, 2001; Mendoza Bertelli et al., 2016). While SA and methicillin-resistant *S. aureus* (MRSA) are recognized as common etiologic agents of osteomyelitis (Urish and Cassat, 2020), there remains work to be done in identifying specific causative sequence types (ST) and clonal complexes (CC) (Pimentel de Araujo et al., 2021). Thorough characterization of the molecular epidemiology of SA involved in osteomyelitis may contribute to understanding of pathogenesis (Post et al., 2014) and strengthen therapeutic strategies, whether those are pre-existing strategies, novel techniques, or combinations thereof.

Along this vein of investigation, SA ST398 has been increasingly recognized as an important human pathogen (Rasigade et al., 2010; Valentin-Domelier et al., 2011; van der Mee-Marquet et al., 2011; Kashif et al., 2019). Previously thought to be primarily a colonizer and occasional pathogen of livestock (de Neeling et al., 2007, Wulf and Voss, 2008), ST398 is now a known causative organism of serious human infections, sometimes carrying methicillin resistance (Armand-Lefevre et al., 2005; Witte et al., 2007; Wulf and Voss, 2008; Schijffelen et al., 2010) and occurring in the absence of contact with livestock (Murra et al., 2019). Human illnesses caused by ST398 include soft tissue infections (Murra et al., 2019), joint infections and osteomyelitis (Post et al., 2014; Senneville et al., 2014; Park et al., 2019), blood-stream infections (BSI) (Valentin-Domelier et al., 2011), and pneumonia, including ventilator-associated pneumonia and lethal, necrotizing pneumonia (Rasigade et al., 2010). Virulence factors and pathogenesis of ST398 are incompletely understood (Smith and Pearson, 2011). Further investigations of CC398 and ST398 in particular are imperative to continue advancing the understanding of Staphylococcal osteomyelitis, with the ultimate goals of enhancing therapeutic strategies, minimizing negative impact to the patient, and reducing overall burden on the healthcare system.

In this work, we describe an investigation into the patho-mechanisms of methicillin-sensitive SA ST398, clinically isolated from a goat suffering hypertrophic osteomyelitis. Our goal was to investigate the pathogenesis, virulence, and interspecies capability of ST398 strain UTCVM1 as an etiologic agent of bacterial osteomyelitis. The ability of ST398 strain UTCVM1 to invade pre-osteoblastic cells and trigger inflammatory responses including apoptosis was investigated *in vitro*. An *in vivo* study was conducted to determine the capability of ST398 to induce osteomyelitis in a rodent mandibular defect model. We hypothesized that ST398 strain UTCVM1 would: 1. be capable of intracellular invasion *in vitro*, 2. induce significant inflammation, apoptosis, and alter osteoregulatory mechanisms, and 3. be capable of inducing osteomyelitis in an *in vivo* rodent model unless faced with a high dose of local antibiotics.

## 2 Materials and Methods

### 2.1 *In vitro* methodology

#### 2.1.1 Bacterial strain selection and preparation

SA ST398 strain UTCVM1 was selected for these experiments as a clinical isolate from a goat suffering hypertrophic osteomyelitis following orthopedic surgery (Abouelkhair et al., 2018). ST398, as described above, is recognized as a colonizer and pathogen of livestock as well as people. This particular isolate is methicillin-sensitive but resistant to other classes of antimicrobials (**Table 1**). SA USA300 (ATCC BAA-1556) is a human origin isolate that produces severe, invasive osteomyelitis in pediatric and adult patients (Peyrani et al., 2012). USA300 is recognized as a highly virulent Staphylococcal strain type (Thurlow et al., 2012) and is often utilized in osteomyelitis research, both *in vitro* (Saleh-Mghir et al., 2012; Strobel et al., 2016) and *in vivo* (Saleh-Mghir et al., 2012; Davido et al., 2016; Sahukhal et al., 2020). SA Cowan1 (ATCC 12598) is a non-cytotoxic human origin septic arthritis isolate (Strobel et al., 2016). Cowan1 is often utilized experimentally as a minimally virulent strain type of SA (Grundmeier et al., 2010).

**Table 1:** Antimicrobial Susceptibility of SA ST398 Isolates.

Antibiotic	ZOI (mm)	Category	MIC (μg/mL)
Penicillin G	N/A	R	4
Clindamycin	6	R	>10
Erythromycin	6	R	>54
Gentamicin	22	S	0.87
Oxacillin	27	S	0.3
Tetracycline	10	R	>33
Rifampicin	N/A	S	<= 1
Vancomycin	N/A	S	<=1

Antimicrobial susceptibility test (AST) results for ST398 strain UTCVM1 utilized for in vitro and in vivo experiments. AST values of recovered ST398 isolates (from two rats) and inoculated ST398 (UTCVM1) did not differ. N/A, Not applicable.

Bacterial inoculums were prepared for cell culture by growing each of the three bacterial strain types overnight in 5mL of tryptic soy both (TSB) at 35°C with aeration (Prescott and Klein, 2002; Cunha, 2005; Skov et al., 2009). Bacteria were harvested by centrifugation (10 minutes at 4300 X *g*), washed twice in 5mL of Hank’s balanced salt solution (HBSS) and resuspended in Minimum Essential Media α (α-MEM) (Thermo Fisher Scientific) with 10% fetal bovine serum (FBS) as described by Tucker et al. (2000).

#### 2.1.2 Cell culture and bacterial inoculation

Commercially obtained MC3T3-E1 subclone 4 cells (ATCC) were utilized for *in vitro* experiments. MC3T3-E1 cells are immature osteoblastic cells of murine origin. Cells were expanded in tissue culture treated polystyrene flasks and incubated under standard conditions (37°C and 5% CO_2_) in α-MEM media with 10% (FBS), 1% penicillin streptomycin (pen-strep) and amphotericin B. Media was changed every 2 to 3 days. Once cells reached approximately 90% confluency, they underwent enzymatic release from the growth substrate utilizing 0.25% Trypsin-EDTA solution for 2 minutes at 37°C (Jackson et al., 2018). Cells were collected and allocated to experimental set-up.

Expanded MC3T3-E1 cells were plated at a density of 1x10^5^/well in 12-well tissue culture-treated plates or 12-well transwell plates (Thermo Fisher Scientific). Cells were maintained in α-MEM media under standard conditions as described above. Media was changed every 2-3 days until cells reached 80% confluence. Three wells of each plate (biologic triplicates) were allocated as uninfected controls, and remaining wells received either ST398, USA300 or Cowan1, all in triplicate. Experimental wells were then infected as previously described by Tucker et al. (2000), with a bacterial inoculum of 10^8^ colony forming units (CFU) per well, which provided a multiplicity of infection (MOI) of 250:1. Post-inoculation, control and experimental wells were incubated under standard conditions for 45 minutes to allow for bacterial invasion of plated cells. Post-inoculation and incubation, supernatant was removed, cells were washed twice with 3mL of HBSS, and 3mL α-MEM media containing 25μg gentamicin (gentamicin sulfate, 100mg/mL) was replaced in wells to eliminate extracellular bacteria.

#### 2.1.3 Apoptosis assay

Following bacterial invasion, control and experimental samples in biologic triplicates were subjected to apoptosis detection utilizing the Pacific Blue™ Annexin V/SYTOX™ AADvanced™ Apoptosis Kit (Thermo Fisher Scientific) for flow cytometric analysis. After 4 and 20 hours of incubation, supernatants were removed and saved from each well. Cells were washed once with 3mL of HBSS, and wash collection was saved with the collected supernatant, as previously described (Tucker et al., 2000). Cells were analyzed by flow cytometry utilizing an Attune flow cytometer. Phosphatidylserine was measured on the surface of apoptotic cells using Pacific BlueTM - conjugated annexin, while dead cells were detected with SYTOXTM AADvancedTM stain.

#### 2.1.4 Inflammatory cytokine analysis

Control and experimental samples in biologic triplicates were used for inflammatory cytokine analysis. Experimental samples were infected as described above (bacterial inoculation) and maintained at 37°C. At 8, 24 and 48 hours post-inoculation, supernatant was collected from each well and saved individually (in duplicate) at -80°C. At these times, samples were washed twice with 3mL of HBSS and fresh media was replaced in wells. Concentration of interleukin-6 (IL-6) was determined by enzyme-linked immunosorbent assay (ELISA) analysis per manufacturer instruction (R&D Systems, Minneapolis, MN. USA). Optical densities of samples and standards were measured at 450nm with an iMark microplate reader (BioRad).

#### 2.1.5 Osteoregulatory cytokine analysis

Control and experimental samples in tissue-culture treated plates in biologic triplicates were dedicated to cytokine analysis to evaluate bone signaling and remodeling. Samples underwent supernatant collection, washing and media replacement immediately following bacterial inoculation, (time 0). Following this sample collection, media was replaced with osteoinductive media (α-MEM media supplemented with 10mM beta glycerophosphate, 10nM dexamethasone and 155μM ascorbic acid) (Bow et al., 2020) that was changed every 2-3 days. Sampling procedure was repeated at days 7, 14 and 21. Supernatant was saved at -80°C. Concentrations of osteoprotegerin (OPG) and osteopontin (OPN) were determined by ELISA analysis per manufacturer instruction (R&D Systems, Minneapolis, MN. USA). Optical densities of samples and standards were measured at 450nm with an iMark microplate reader (BioRad).

#### 2.1.6 Transmission Electron Microscopy

Samples seeded on the transwell plate were dedicated to transmission electron microscopy (TEM) following 45 minute bacterial inoculation. Ninety minutes after bacterial inoculation (Tucker et al., 2000), cells were fixed in cacodylate buffered (.05M) glutaraldehyde (3%) for one hour, washed three times in buffer and then post-fixed in 2% buffered osmium tetroxide for one hour. After post-fixation, samples were washed in water three times over 30 minutes and then dehydrated in an ethanol gradient (10%, 25%, 50%, 75%, 95%, 100%) 15 minutes per step, with two final steps in 100% propylene oxide. Following dehydration, samples were embedded in epoxy (EMBed 812) using the following routine:

EMBed 812: Propylene oxide 1:3 (two hours)
EMBed 812: Propylene oxide 3:1 (overnight)
EMBed 812 100% (three changes over 8 hours)

Samples were then placed in fresh EMBed 812 in flat embedding molds and the epoxy was cured overnight in an oven at 68° C. Ultrathin sections (~100nm) were cut on a Leica UC7 ultra-microtome using a diamond knife (Diatome). Sections were mounted on copper grids and stained with 50% methanolic uranyl acetate (45min) washed in water and post-stained with Reynolds lead citrate (5minutes), washed and completely dried. Sections were imaged with a JEOL 1400-Flash (JEOL, USA) operating at 120kV. Images were recorded with a GATAN One View camera.

### 2.2 *In vivo* challenge

#### 2.2.1 Rat mandibular defect model

Twenty-four, 8-week-old, male, outbred Wistar rats (Charles River Laboratories, USA) weighing 265 ± 23.45g were used in this study. Animals were housed and cared for according to Institutional Animal Care and Use Committee (IACUC) guidelines. All procedures were approved by IACUC as well as the Institutional Biosafety Committee (IBC). Animals were maintained in laboratory space approved for biosafety level 2 (BSL-2) procedures under standard conditions with free choice rodent chow and water.

SA ST398 strain UTCVM1 was utilized as a sole bacterial organism to induce osteomyelitis in a rat mandibular defect model. It was maintained on blood agar at 4°C throughout the study period. Thirty-six hours prior to surgical inoculation, one bacterial colony was streaked onto a blood agar plate and placed in an aerobic incubator at 35°C. Four hours prior to surgical inoculation, 1-3 colonies were isolated, placed in TSB and incubated for three hours at 35°C with aeration. After three-hour incubation, bacterial broths were diluted to 10^8^ CFU/mL in sterile saline. This CFU count was equivalent to a McFarland standard of 0.5.

Bacterial osteomyelitis was induced using a procedure adapted from Sodnom-Ish et al. (Sodnom-Ish et al., 2021). Briefly, the procedure operated under the premise of creating bone trauma, introducing a high dose of planktonic bacteria locally, and placing foreign material within traumatized bone to provide a nidus for bacterial colonization. This procedure fulfills the main tenants of osteomyelitis induction in animal models (Guarch-Pérez et al., 2021; Billings and Anderson, 2022). All procedures were performed in a biosafety cabinet in adherence to BSL-2 safety protocols. Rats were anesthetized using anesthetic gas (isoflurane) and administered a 0.03mg/kg dose of buprenorphine subcutaneously (SQ). They were maintained on isoflurane vaporized into 100% oxygen *via* nose cone throughout the surgical preparation and procedure. Surgical preparation consisted of shaving the fur from the right hemimandible, and skin was aseptically prepared utilizing povidone iodine and isopropyl alcohol. During surgery, skin and muscle layers were sharply incised to expose the right hemimandible. A 3mm circular defect was created utilizing a 3mm diameter carbide round drill bit (Stryker Instruments, Kalamazoo, MI. USA) attached to a handheld microdrill (Ideal Microdrill, CellPoint Scientific, Inc. Gaithersburg, MD. USA). Defect placement was on the right mandibular ramus, caudal to tooth roots and ventral to pulp cavity. Once the defect was created, a 3x2mm circular wafer of sterile, commercially available bovine-origin bone mineral replacement material (Geistlich Bio-Oss^®^ Collagen, Geistlich Pharma AG, Wolhusen, Switzerland) was placed into the defect site. Rats were allocated into one of four groups, as described in **Table 2**. Muscle and skin incisions were closed with 4-0 poliglecaprone 25 (Monocryl, Ethicon, Inc. Raritan, NJ. USA) suture in an interrupted cruciate pattern.

**Table 2:** Experimental Groups.

Group, Size	Bone Defect (Y/N)	Bio-Oss Collagen (Treatment Type)	Bacterial Inoculum
I	Y	pre-loaded with 2.25mg vancomycin	none
II	Y	pre-loaded with 2.25mg vancomycin	10^8^ CFU ST398 suspension, 50μL
III	Y	pre-loaded with 1.12mg vancomycin	10^8^ CFU ST398 suspension, 50μL
IV	Y	native - no antimicrobial loaded	10^8^ CFU ST398 suspension, 50μL

Once recovered from anesthesia, animals were provided free choice water and soft gel diet. Buprenorphine at 0.03mg/kg was administered SQ every 12 hours for the first three days following surgery. At day five post-operatively, animals were transitioned from the soft gel diet to regular rodent chow. Animals were maintained for four weeks with once weekly venipuncture, weighing, and surgical site inspection. At four weeks, they were sacrificed *via* anesthetic overdose (isoflurane) and thoracotomy. At the time of sacrifice, gross necropsies were performed. Right hemimandibles were harvested under aseptic conditions. During harvest, cultures were obtained from bone defects, bordering soft tissues, and any other apparent lesion present. Hemimandibles were formalin fixed in 10% neutral buffered formalin (NBF) and micro-computed tomography (micro-CT) was performed, followed by histological analysis.

#### 2.2.2 Material description

Bio-Oss^®^ Collagen was utilized as dual drug delivery device and scaffold material to fill space and serve as a potential nidus for bacterial colonization within the defect site. Bio-Oss^®^ is a biocompatible bone mineral matrix composed of 90% purified, bovine-derived small bone particles and 10% porcine collagen (AG GP, 2013). Bio-Oss^®^ Collagen is provided as a sterilized block of varying sizes that can be trimmed into desired size and shape either dry or moistened. There are interconnected pores that extend throughout the material which contribute to hydrophilicity of the device, and allow for simple hydration of the matrix (AG GP, 2013). This material is labeled and utilized for defect-filling and guided bone regeneration (GBR) (Galindo-Moreno et al., 2010; AG GP, 2013).

#### 2.2.3 *In vitro* drug loading and elution

To determine the loading capacity of Bio-Oss^®^ Collagen for vancomycin, *in vitro* hydrophilicity and drug elution experiments were performed. Material was trimmed into 3x2mm circular wafers utilizing a 3mm biopsy punch and sharp dissection with a #10 blade. Hydrophilic properties of the material were determined by calculating the percent equilibrium water content (EWC) (equation 1). Phosphate buffered saline (PBS) was added in 5μL increments until the devices (n=3) were saturated and wet weights were recorded.

Equation 1:


$$EWC\;\left(\%\right)=\;\frac{\left(Weight\;hydrated\;sample-weight\;dry\;sample\right)}{Weight\;hydrated\;sample}\;x\;100$$

Based on the determined hydrophilic properties and the size of 3x2mm, the optimal loading volume of vancomycin (50mg/mL) was determined to be 45μL.

Bio-Oss^®^ Collagen wafers were loaded with either 2.25mg (high dose, n=3) or 1.12mg (low dose, n=3) of vancomycin. Vancomycin dose of 2.25mg was accomplished by loading 45μL of 50mg/mL vancomycin solution. To ensure equal loading volume across devices, the 1.12mg dose was accomplished by loading 45μL of 25mg/mL vancomycin. The lower concentration was created by diluting 50mg/mL vancomycin 1:1 with sterile water for injection. Devices were loaded and allowed to incubate at room temperature for four hours prior to use in the elution experiment.

At four hours post-loading, devices were placed into sterile polystyrene plates in individual wells with 2mL PBS/well. Devices were incubated at 37°C to mimic physiologic temperature. At pre-determined timepoints (3, 12, 24 and 48 hours, and on days 4, 6, 8, 10, 12 and 14), supernatant was removed, saved at -80°C and 2mL of PBS was replenished in each well prior to returning to incubation.

#### 2.2.4 Analytical chemistry

Analysis of vancomycin in phosphate buffered saline (PBS) samples was conducted using reversed phase HPLC (University of Tennessee College of Veterinary Medicine Pharmacology Laboratory). The system consisted of a 2695 separations module and a 2487 UV detector (Waters, Milford, MA, USA.). Separation was attained on a Waters XBridge C_18_ 4.6 x 250 mm (5 µm) column protected by a 5 µm XBridge C_18_ guard column. The mobile phase was an isocratic mixture of 0.1% formic acid in water pH 3.0 with 2 M NaOH and acetonitrile (90:10). It was prepared fresh daily using double-distilled, deionized water filtered (0.22 μm) and degassed before use. The flow rate was 1.0 ml/min and UV absorbance was measured at 240 nm.

Vancomycin was extracted from PBS samples using a precipitation method. Briefly, previously frozen PBS samples were thawed and vortexed and 100 µL was transferred to a screw-top test tube followed by 15 µL internal standard (100 µg/mL caffeine). Five hundred microliters of acetonitrile was added and the tubes were vortexed for 30 seconds and then centrifuged for 10 minutes at 1000 X *g*. The organic layer was transferred to a 16x100 mm tube and evaporated to dryness with nitrogen gas. Samples were reconstituted in 250 µL of mobile phase and 100 µL was analyzed.

Standard curves for PBS analysis were prepared by fortifying PBS with vancomycin to produce a linear concentration range of 0.1–2000 µg/mL Calibration samples were prepared exactly as PBS samples. Average recovery for vancomycin was 95% while intra and inter-assay variability ranged from 1.6 to 4.5% and 2.2 to 7.9%, respectively. The lower limit of quantification was 0.1 µg/mL.

#### 2.2.5 Cytokine expression analysis

RNA was extracted from whole blood samples collected at predetermined timepoints (day 0, weeks 1, 2, 3 and 4) utilizing RNeasy mini kits (Qiagen, Hilden, Germany). Quantity and quality of extracted RNA was assessed *via* NanoDrop Spectrophotometer and samples were saved at -80°C until polymerase chain reaction (PCR) analysis. Real-time PCR was performed to assess IL-6, IL-1a, RANKL and sclerostin (*sost*) genes expression utilizing TaqMan^™^ Gene Expression Assays (Thermo Fisher Scientific) for IL-6, IL-1a, RANKL, and *sost*, respectively, with GAPDH (Thermo Fisher Scientific) as a housekeeping gene. Assays were run on QuantStudio^™^ 3 Real-Time PCR System (Thermo Fisher Scientific) in technical duplicates. Cycle threshold (Ct) values from technical duplicates were averaged together. Ct values from day 0 were utilized as a baseline for each animal and gene, and the delta-delta Ct (ΔΔCt) method was used to determine relative fold gene expression through time.

#### 2.2.6 SA recovery and analysis

Cultures obtained from bone and bordering soft tissues following study termination at four weeks post-operatively were submitted for aerobic and anaerobic culture and observed each day for five days. The following agar plates were inoculated: Columbia Blood agar with 5% sheep blood and Columbia NaladixicAcid (CNA) with 5% sheep blood agar (35°C, 5% CO_2_), MacConkey II (35°C, ambient), CDC and Phenylethyl alcohol (PEA) (35°C, anaerobic). Isolated colonies were then Gram stained and identified *via* matrix-assisted laser desorption/ionization coupled with time-of-flight mass spectrometry (MALDI-TOF) (Bruker). ASTs for SA isolates were performed using the Kirby-Bauer technique (KB) and repeated *via* minimum inhibitory concentration (MIC) (ThermoFisher Scientific). For KB overnight subculture was diluted in 0.85 saline to match the turbidity of a 0.5 McFarland standard. The dilution was swabbed on a Mueller-Hinton (MH) plate, allowed to dry for 10-15 minutes, and antimicrobial discs were applied, all per Clinical and Laboratory Standards Institute guidelines (CLSI, 2018). Plates were incubated for 24 hours at 35°C. Zones of inhibition (ZOI) were measured (mm) and documented as susceptible, intermediate or resistant (S/I/R). To measure MIC following CLSI Vet 01, overnight subculture was diluted in sterile water to match the turbidity of a 0.5 McFarland standard; 30μL added to 11.0 ml MH broth; 50μL added to each well of COMPGP plate and incubated for 24 hours at 35°C. MIC results were read using BioMic V3 (Giles Scientific USA). Additionally, whole genome sequencing (WGS) was performed on recovered SA isolates to determine individual isolate characteristics as previously described (Abouelkhair et al., 2018).

Following culture, DNA extraction was performed on culturette swabs utilizing DNeasy kits (Qiagen, Hilden, Germany). Quantity and quality of DNA was assessed *via* NanoDrop Spectrophotometer and samples were saved at -20°C. PCR analysis (qPCR) was performed to investigate presence of SA utilizing TaqMan™ *Staphylococcus aureus* Detection Assay according to the University of Tennessee College of Veterinary Medicine Immunology and Virology Laboratory procedure. Samples were run in technical duplicates on the QuantStudio^™^ 3 Real-Time PCR System (Thermo Fisher Scientific). Ct values from technical duplicates were averaged together and values were interpreted to be positive or negative for the presence of SA DNA from culturette swabs.

Whole genome sequencing (WGS) was performed on recovered SA isolates. Sequencing libraries were constructed using the Nextera DNA sample prep kit (Illumina, Inc., USA) according to the manufacturer’s instructions. Paired end, 150 bp reads were generated on an illlumina MiniSeq platform (Illumina, Inc.) at the Virology Diagnostic Laboratory, University of Tennessee. Sequences were trimmed using BBDuk and *de novo* assembled using Geneious prime version 11.0.14. Annotation was performed by the NCBI Prokaryotic Genome Annotation Pipeline version 6.3 (https://www.ncbi.nlm.nih.gov/genome/annotation_prok) using the best-placed reference protein set with GenMarkS+.

#### 2.2.7 Micro-computed tomography

Right hemimandibles were imaged by the University of California, Davis Veterinary Orthopedic Research Laboratory using a micro-CT specimen scanner (μCT 35, Scanco Medical; Bassersdorf, Switzerland). Scan parameters were 55kVp, 145μA, 300msec exposure time, average of 3 exposures per projection, 0.5 mm aluminum filter, 500 projections per 180 degrees and a 10 micron voxel size. The raw images were calibrated using a hydroxyapatite (HA) phantom of varying HA concentrations. Noise in the images was reduced by use of a low-pass Gaussian filter. A threshold of 380mgHA/cc was used to partition mineralized tissue from other less-dense tissues. Bone volume fraction (BV/TV) was determined by dividing the number of voxels more dense than the threshold representing mineralized tissue (BV: bone volume) by the total number of pixels in the region (TV: total volume). The mean density of all material in the volume is apparent bone mineral density (aBMD). The mean density of only the mineralized material is the tissue mineral density (TMD).

#### 2.2.8 Histological analysis

Excess soft tissue was trimmed from formalin-fixed hemimandibles (if applicable). Hemimandibles were decalcified in Formical-2000 for 48 hours until they could be sharply dissected with minimal resistance. After decalcification, hemimandibles were transferred to 10% NBF and submitted for histology along with recovered soft tissue (University of Tennessee College of Veterinary Medicine, Veterinary Diagnostic Laboratory, Histopathology Service). Tissues were embedded within paraffin, 4μm decalcified sections were obtained and stained with Hematoxylin and Eosin (H&E). Slides were evaluated qualitatively and scored in a binary fashion, recording the presence or absence of osteomyelitis. Osteomyelitis was characterized by suppurative or degenerative neutrophilic inflammation, inflammatory infiltrates into bone tissue, and bone destruction or necrosis.

## 3 Statistical Analysis

IL-6 ELISA Data: The effects of strain types, time and their interaction on IL-6 concentration were evaluated using mixed model analysis for repeated measures with time as the repeated factor. Rank data transformation was applied when diagnostic analysis on residuals exhibited violation of normality and equal variance assumptions using Shapiro–Wilk test and Levene’s test. *Post hoc* multiple comparisons were performed with Tukey’s HSD. Statistical significance was identified as p-values (alpha-error) at< 0.05. Analyses were conducted in SAS 9.4 TS1M7 (SAS institute Inc., Cary, NC). Vancomycin elution data: The effects of treatment, dose and time on response variable total vancomycin were examined using mixed model analysis for repeated measures. Ranked transformation was applied when diagnostic analysis on residuals exhibited violation of normality and equal variance assumptions using Shapiro–Wilk test and Levene’s test. *Post hoc* multiple comparisons were performed with Tukey’s adjustment. Statistical significance was identified as p-values (alpha-error) at< 0.05. Analyses were conducted in SAS 9.4 TS1M4 (SAS institute Inc., Cary, NC). PCR cytokine data: effects of group and week on delta-delta CT of the genes were analyzed using repeated measures ANOVA respectively, with group as the between-subject factor while week as the repeated factor. Diagnostic analysis was conducted to exam model assumptions. Ranked transformation was applied if diagnostic analysis exhibited violation of normality and equal variance assumptions. *Post hoc* multiple comparisons were performed with Tukey’s adjustment. Statistical significance was identified at the level of 0.05. Analyses were conducted in SAS 9.4 TS1M7 for Windows 64x (SAS institute Inc., Cary, NC).

Micro-CT data: Two-way mixed effect ANOVA model was utilized to analyze the effect of treatment, ROI and the treatment by ROI interaction on TV, BV, BV/TV ratio, apparent bone mineral density, and tissue bone mineral density, with treatment as the between subject factor and ROI as the within subject factor (SAS 9.4, PROC GLIMMIX). The least square means computed and separated using the Fisher Least Significant Difference (LSD) Method. A Shapiro-Wilk W and QQ normal plot were used to evaluate the normality of the ANOVA residuals. The variable TV was taken rank transformation because of non-normality of TV. P<0.05 was considered as significant. SAS Institute Inc., Cary, NC. version 9.4, release TS1M7 was used for all analysis.

## 4 Results

### 4.1 *In vitro* characterization

#### 4.1.1 Apoptosis assay

**Figure 1:**
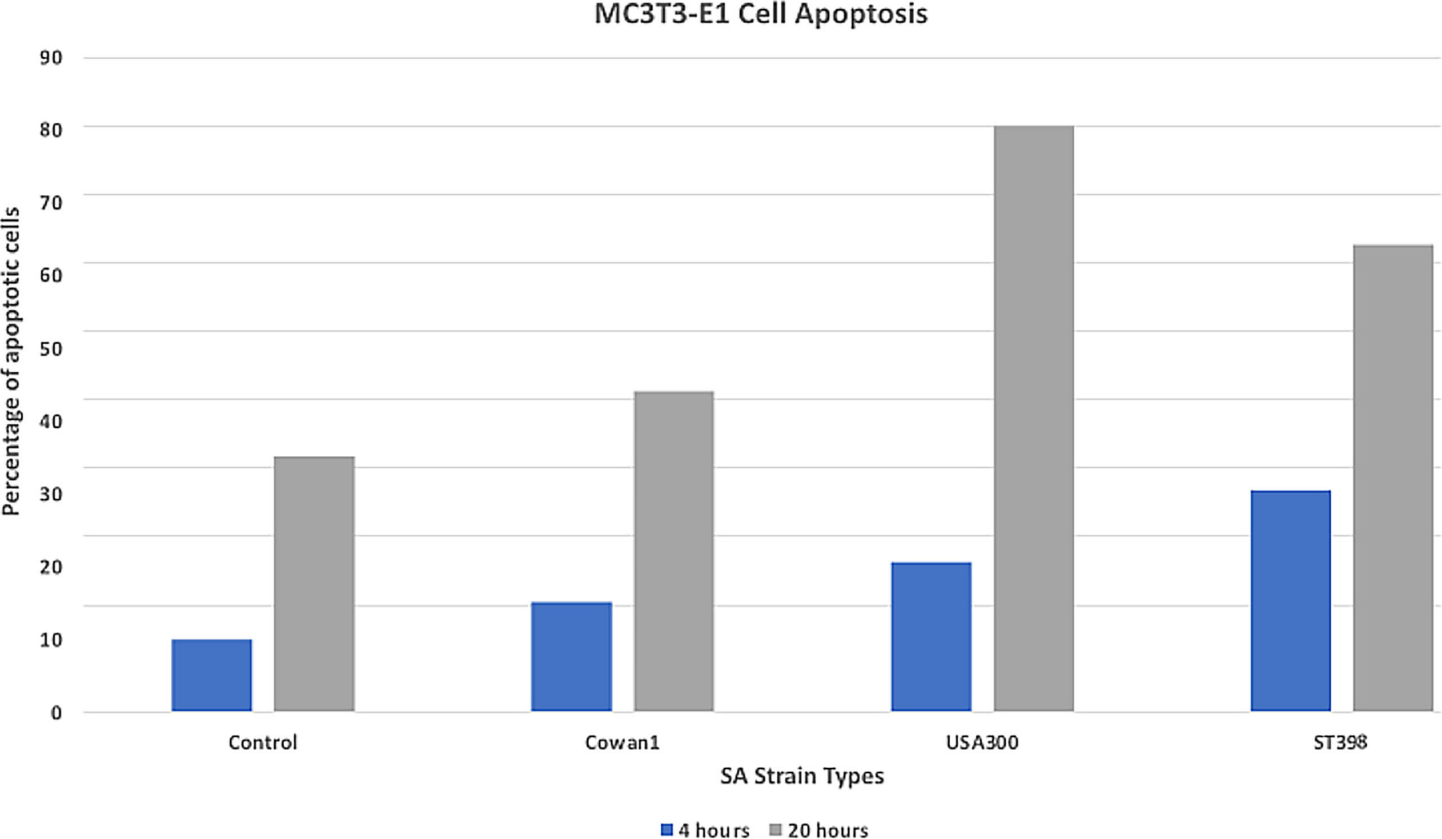
Apoptosis assay. Flow cytometry annexin staining for early (4hr) and late (20hr) apoptosis. USA300 appeared to induce a stronger apoptotic affect compared with ST398 strain UTCVM1. Cells infected with Cowan1 underwent apoptosis to a lesser extent. Uninfected cells served as the control.

Death induction of bone cells is an important feature of SA osteomyelitis (Josse et al., 2015). We measured apoptosis to better understand the *in vitro* cytotoxicity of selected SA strains. Results (**Figure 1**) are reported as mean percent of cells undergoing apoptosis when counting 10,000 cell events. USA300 appeared to induce a more severe apoptotic effect (22.1% at 4hr, 80.6% at 20hr) compared to ST398 (30% at 4hr, 64.2% at 20hr). Cells infected with Cowan1 underwent apoptosis to a lesser extent (15% at 4hr, 44.1% at 20hr). Uninfected cells acted as a control (9% at 4hr, 35% at 20hr).

#### 4.1.2 Inflammatory cytokine analysis

**Figure 2:**
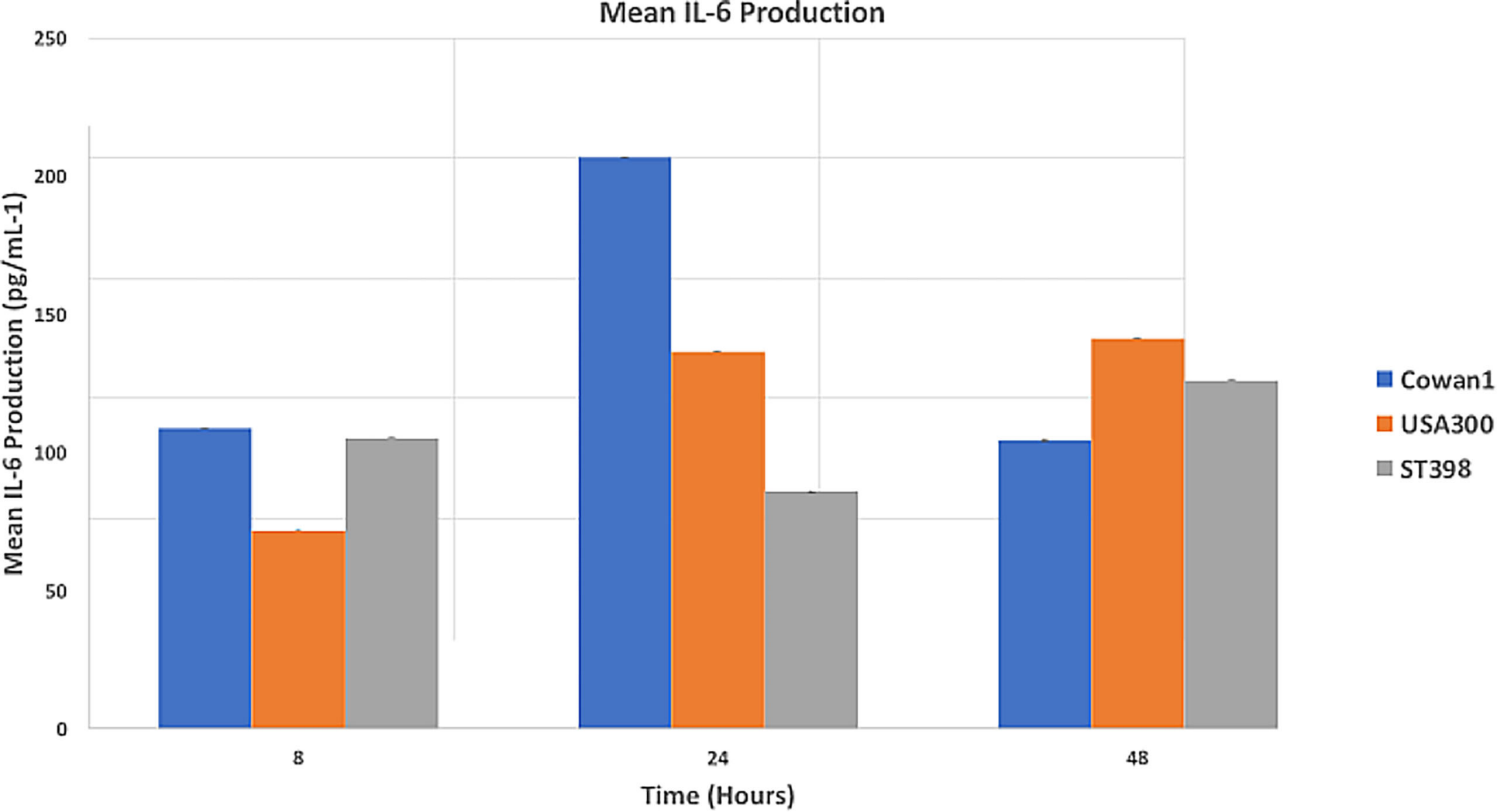
Mean IL-6 Production. MC3T3-E1 cells were inoculated with SA ST398, USA300 or Cowan1. Cowan1 showed an initial strong inflammatory response that lessened at 48 hours. USA300 became stronger over time. ST398 showed an ebb followed by an increase in inflammatory response. IL-6 expression by Cowan1 at 24 hours was significantly greater than IL-6 expression at 24 hours by ST398. No other significant differences were detected.

Results (**Figure 2**) are reported as mean IL-6 concentration (pg/mL^-1^). Cowan1 infection initiated a strong initial inflammatory response that ebbed by 48 hours (108.84 at 8hr, 206.89 at 24hr, and 108.84 at 48hr). USA300 showed an initial increase in inflammation that slightly decreased (71.75 at 8hr, 136.6 at 24hr, 104.31 at 48hr). ST398 showed inflammation that initially decreased followed by a marked increase by the end of the study period (105.17 at 8hr, 85.89 at 24hr, and 125.98 at 48hr). There was a significant difference between Cowan1 and ST398 at 24 hours, with Cowan1 demonstrating greater concentrations of IL-6 (p=0.0342). Otherwise, no significant differences were detected.

#### 4.1.3 Osteoregulatory cytokine analysis

Results are reported as mean OPN and OPG concentration (pg/mL^-1^). Control samples expressed OPN and OPG at consistent levels throughout the study period (OPN: 3851.96 at 0d, 3906.41 at 7d, 2643.44 at 14d, and 3920.3 at 21d, OPG: 2069.09 at d0, 2832.12 at d7, 2093.18 at d14, and 2592.58 at d21). Cowan1 infection expressed OPN and OPG only on day 0 (3887.7, 2166.21). USA300 infection expressed OPN and OPG only on day 0 (3898.81, 2107.42). ST398 infection expressed OPN and OPG only on day 0 (3878.63, 2185).

#### 4.1.4 Transmission electron microscopy

**Figure 3:**
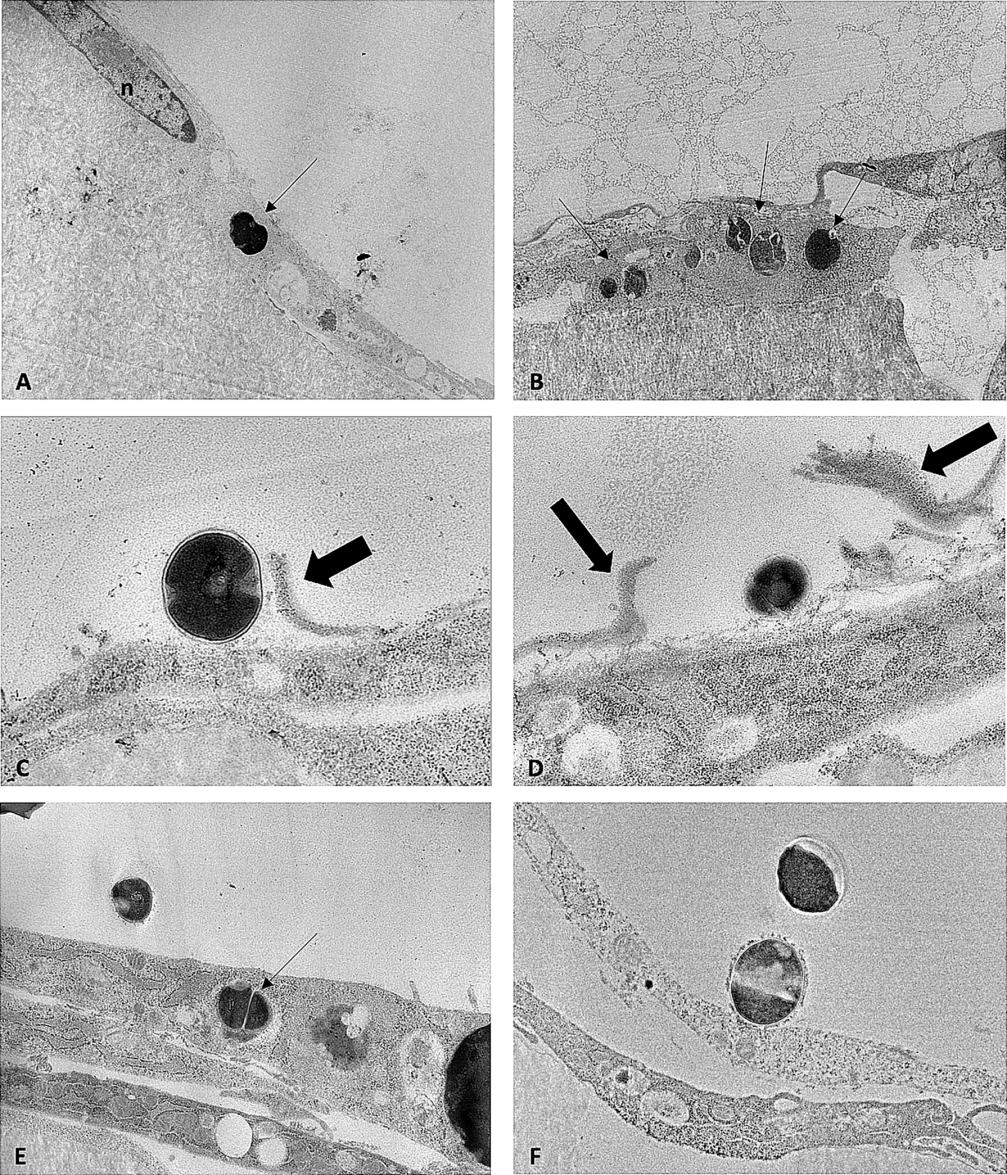
TEM images obtained from MC3T3-E1 cells infected with SA Cowan1 **(A)**, USA300 **(B–D)**, and ST398 **(E, F)**. **(A)**: single intracellular Cowan1 organism (black arrow), distorted in shape. **(B)**: one osteoblastic cell infected with multiple USA300 organisms (black arrows). **(C, D)**: USA300 organisms bordering osteoblastic cells. Thick black arrows denote discontinuous cellular membranous material which may indicate incorporation of USA300 into the cell by filipodia or may indicate disrupted cellular membranes. **(E)**: one intracellular and one extracellular ST398 organism. **(F)**: extracellular ST398 organisms. One ST398 bordering an osteoblastic cell, surrounded by a halo of membranous material. Scale bar = 0.7μm.

Proportions of intracellular and extracellular SA differed between strain types (**Figure 3**). Additionally, morphologic differences were observed between SA sequence types within infected pre-osteoblastic cells. Cowan1 appeared weakly infective, with few intracellular bacteria and no observed extracellular bacteria. Intracellular Cowan1 displayed distorted shapes. USA300 appeared to be highly infective, with many infected pre-osteoblastic cells. Many infected cells contained more than one bacterial organism. Extracellular USA300 was observed. There was also evidence of USA300 bacterial organisms along interrupted pre-osteoblastic cellular membranes. These likely represent extension of filipodia to incorporate USA300 into the cell (Guarch-Pérez et al., 2021) as well as cell rupture. ST398 bacterial organisms with undisturbed morphology were observed within pre-osteoblastic cells, confirming the ability of ST398 to establish intracellular infection. No infected cell contained more than one bacterial organism. Extracellular ST398 was observed. Many extracellular ST398 were in contact with pre-osteoblastic cellular membrane and were encircled by membranous material.

#### 4.1.5 Vancomycin elution from Bio-Oss^®^ collagen, *in vitro*

Bio-Oss^®^ Collagen (3x2mm wafers) were able to load 45μL of vancomycin, (25 or 50mg/mL). Wafers loaded with 1.12mg and 2.25mg of vancomycin eluted vancomycin at concentrations above 0.1μg/mL for 24 hours, at which point concentrations dropped below the limit of quantification (0.1μg/mL) and were not detectable from day 4 (1.12mg) and day 6 (2.25mg) onwards (**Supplementary Materials**). Bio-Oss^®^ Collagen loaded with 1.12mg of vancomycin (n=3) eluted an average of 0.27 ± 0.02mg of vancomycin over a 24-hour period with peak elution of 0.22 ± 0.04mg occurring at three hours. This peak elution is equivalent to eluting 19.77 ± 0.004% of the loaded vancomycin within the first three hours. Bio-Oss^®^ Collagen loaded with 2.25mg of vancomycin (n=3) eluted an average of 0.38 ± 0.02mg of vancomycin over a 24-hour period with peak elution of 0.29 ± 0.01mg occurring at three hours. This peak elution is equivalent to eluting 13.32 ± 0.47% of the loaded vancomycin within the first three hours.

### 4.2 *In vivo* challenge

#### 4.2.1 Animal observation and care

One anesthetic complication occurred and resulted in the death of one rat. Remaining animals (n=23) recovered well from surgery. Adjusted for the loss of one animal, group sizes were as follows: Groups I, II and IV, n=6, Group III, n=5. In the first four days following surgery, animals experienced variable degrees of soft tissue swelling localized to the right mandible. Animals in Group I experienced mild to moderate focal swelling while animals in Group IV experienced moderate to severe swelling generalized to the right mandible. In two animals (rats 21 and 23), the severe, generalized soft tissue swelling to the right mandible caused substantial blepharospasm of the right eye. These animals had mild soft tissue swelling that persisted throughout the study period. They also developed circular areas (2-3mm diameter) of erythema, alopecia and moisture on the skin overlying the surgical site. Throughout the study period, animals ate and drank well. With the exception of animals experiencing severe and generalized swelling, animals appeared subjectively comfortable post-operatively. Once the severe swelling resolved, those animals appeared comfortable as well. Objectively, animals gained weight throughout the study and interacted appropriately with their environment.

#### 4.2.2 SA recovery and characterization

SA was identified in four rats (Group IV, n=3; Group III, n=1) by *in vitro* culture or PCR analysis. Microbiologic cultures yielded positive SA cultures from the bone of two animals from Group IV (rats 18 and 21), along with incidental organisms, including: *Escherichia coli, Staphylococcus xylosus*, and *α-Streptococcus.* PCR analysis demonstrated the presence of SA in the bone of two animals from Group IV (rats 21 and 23) and one from Group III (rat 14), and the soft tissues of two animals from Group IV (rats 21 and 23). This work used the SA MLST database at the University of Oxford (https://pubmlst.org/saureus) (Solyman et al., 2013) for whole genome multilocus sequence typing. Both SA isolates recovered from microbiologic culture were ascribed to ST398 as described previously (Abouelkhair et al., 2018). Ultimately, four (23.5%) out of 17 inoculated rats developed clinical, histologic or microbiologic evidence of SA ST398 osteomyelitis. Of the four SA+ rats, one was in Group III, having been inoculated with SA in the presence of low dose vancomycin (one out of five Group III rats; 20%); three were in Group IV, having been inoculated with SA without antibiotics (three out of six Group IV rats; 50%). The recovered SA had identical antimicrobial susceptibility profiles to the inoculated SA (**Table 1**).

#### 4.2.3 Cytokine analysis

**Figure 4:**
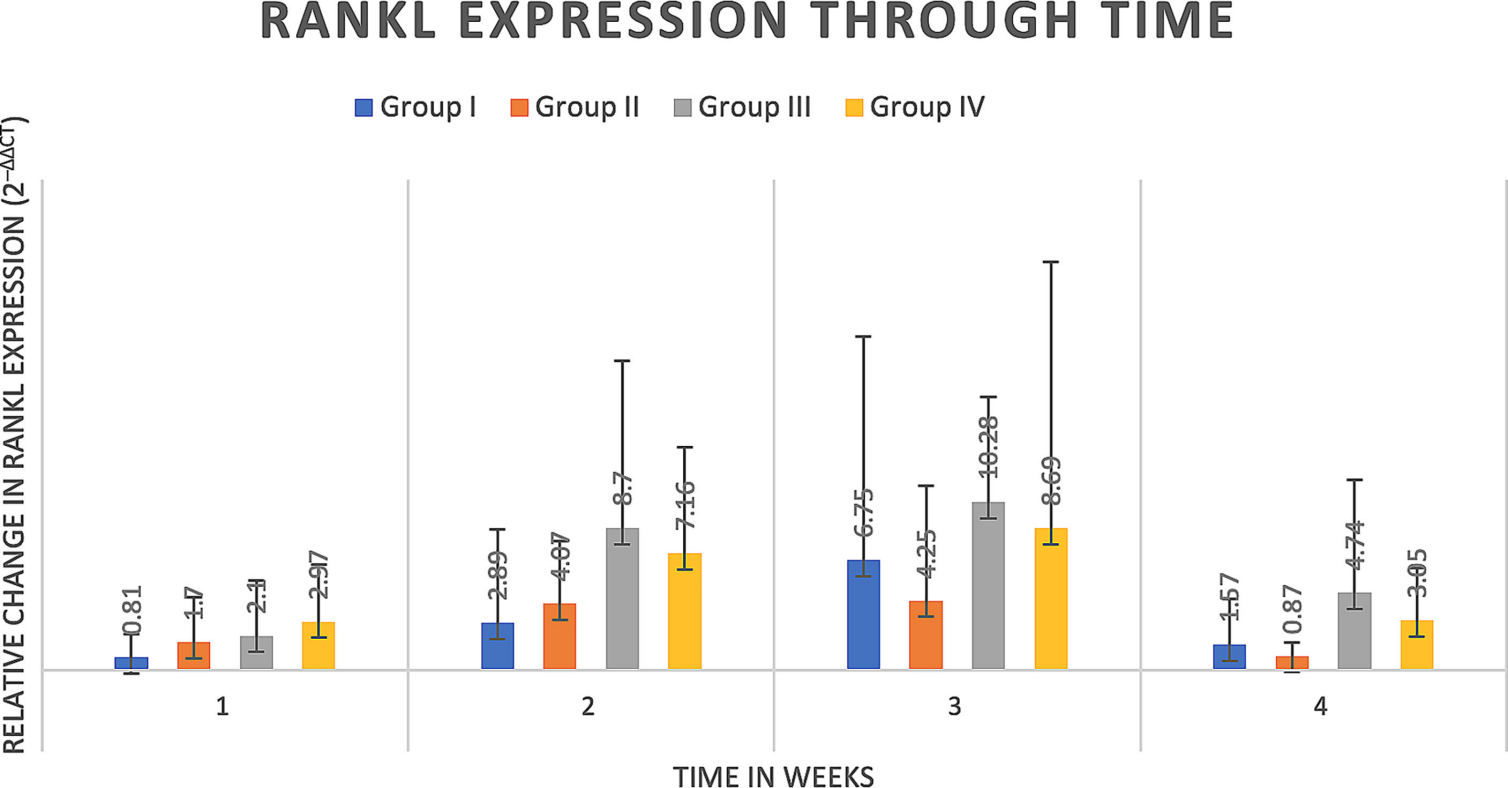
RANKL expression through time expressed as relative fold gene expression. RANKL expression trends higher at weeks two and three and declines at week four. No significant differences were detected between groups through time.

**Figure 5:**
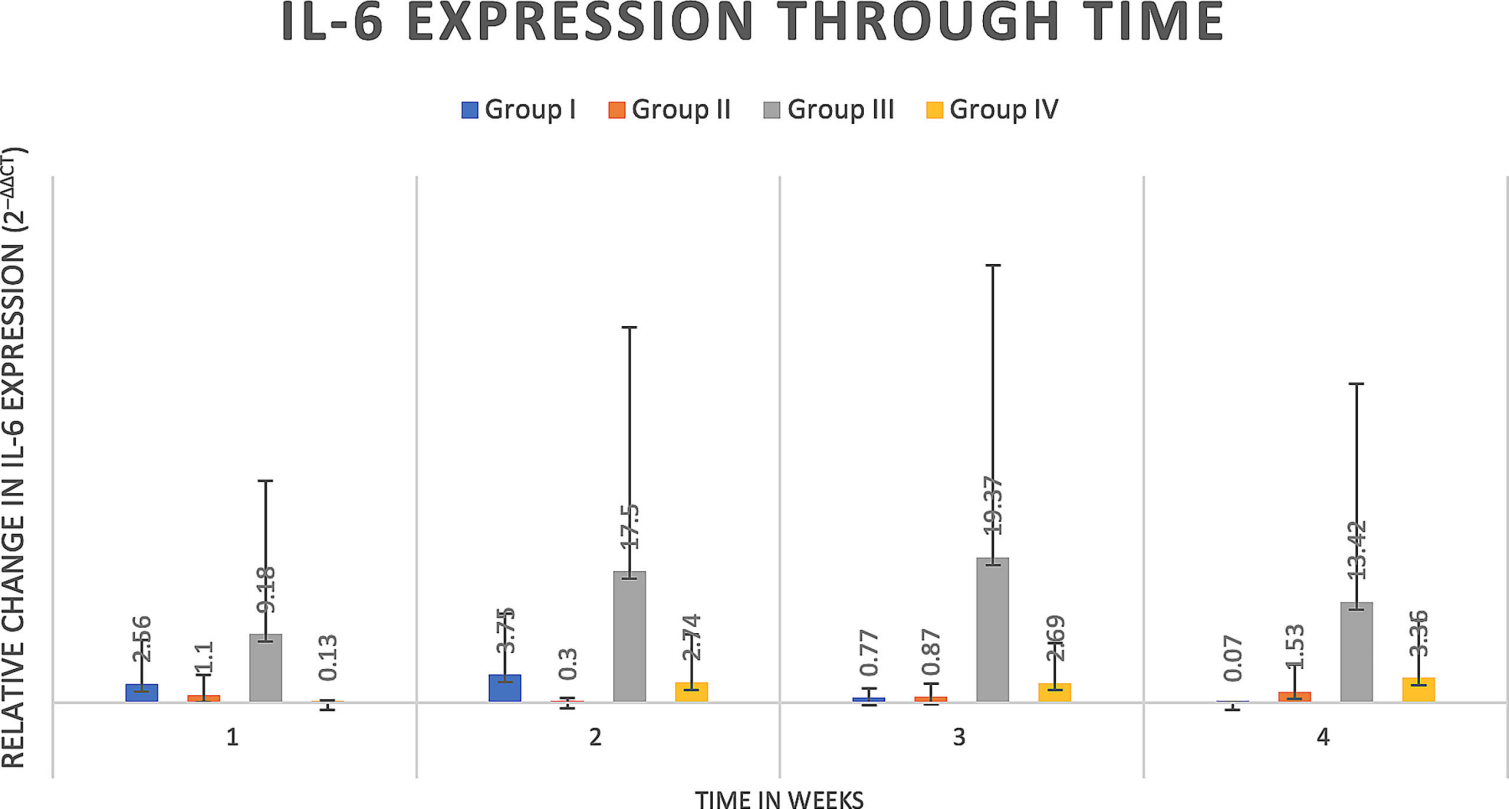
IL-6 expression through time expressed as relative fold gene expression. IL-6 expression follows a similar trend to RANKL expression, trending higher at weeks two and three and declining at week four. Data appears skewed as a result of Group III (rat 14) that had greater IL-6 expression at all timepoints although between groups there were no significant differences detected.

**Figure 6:**
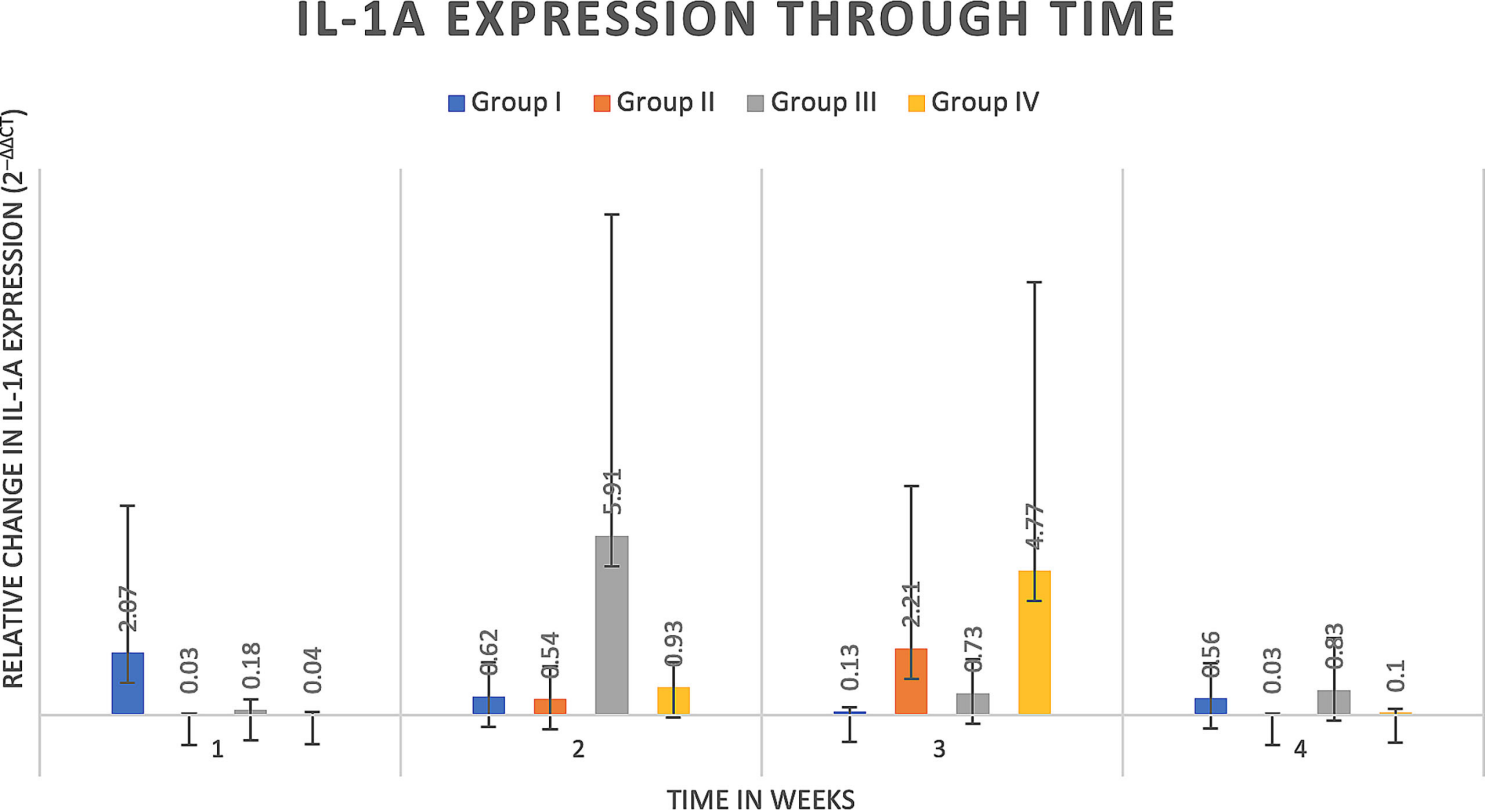
IL-1a expression through time expressed as relative fold gene expression. IL-1a expression in Group IV was significantly greater at week three compared to week one (p=0.0003) and significantly greater at week three than week four (p=0.0228).

Delta delta Ct values were utilized to determine relative fold gene expression through time. Sclerostin (*Sost*) was poorly expressed across groups and throughout time. RANKL expression (**Figure 4**) appeared to increase at weeks two and three and decrease at week four in all groups except the Group I, where RANKL expression did not appear to vary through time. IL-6 expression (**Figure 5**) appeared to follow a similar pattern to RANKL expression, increasing at weeks two and three and decreasing again at week four although there were no significant differences noted between groups, through time. IL-1a expression (**Figure 6**) in Group IV was significantly greater at week three compared to week one (p=0.0003) and significantly greater at week three than week four (p=0.0228).

#### 4.2.4 Micro-CT analysis

Micro-CT images, 30 days after creation of the bone defect, demonstrated consistent defect placement with variations in defect size and bone erosion, most prominent within Groups II-IV. Group III had significantly greater apparent bone mineral density (aBMD) compared to Groups I and IV (p=0.009 and p=0.0467). For remaining parameters of total bone mineral density (tBMD), bone volume (BV) and total volume (TV) there were significant differences between intact and defect bone (p=0.0002, p<0.0001, and p<0.0001, respectively), but no significant differences between treatment groups (**Table 3**).

**Table 3:** Micro-CT Comparison of Intact and Defect Bone.

Defect Bone	aBMD [mg HA/ccm]	tBMD [mg HA/ccm]	BV (mm^3^)	BV/TV
**Group I**	2372.40 ± 610.0	6677.54 ± 267.9	4.52 ± 1.47	0.32 ± 0.10
**Group II**	1847.43 ± 735.03	6819.92 ± 626.35	3.36 ± 1.78	0.24 ± 0.12
**Group III**	2408.62 ± 821.36	6779.74 ± 741.55	4.54 ± 1.88	0.32 ± 0.13
**Group IV**	2022.78 ± 728.32	6478.24 ± 839.95	3.93 ± 1.71	0.28 ± 0.12
**Intact Bone**	**aBMD [mg HA/ccm]**	**tBMD [mg HA/ccm]**	**BV (mm^3^)**	**BV/TV**
**Group I**	2267.36 ± 389.49	5926.12 ± 199.04	1.32 ± 0.27	0.32 ± 0.06
**Group II**	3070.02 ± 1519.49	6290.22 ± 656.75	1.73 ± 0.80	0.43 ± 0.20
**Group III**	3459.6 ± 1151.21	6448.25 ± 422.55	1.98 ± 0.16	0.49 ± 0.16
**Group IV**	2637.06 ± 620.20	5844.45 ± 325.13	1.59 ± 0.38	0.39 ± 0.09

Group III had significantly greater apparent bone mineral density (aBMD) compared to Groups I and IV (p=0.009 and p=0.0467). For remaining parameters of total bone mineral density (tBMD), bone volume (BV) and total volume (TV) there were significant differences between intact and defect bone (p=0.0002, p<0.0001, and p<0.0001, respectively), but no significant differences between treatment groups.

#### 4.2.5 Histological analysis

**Figure 7:**
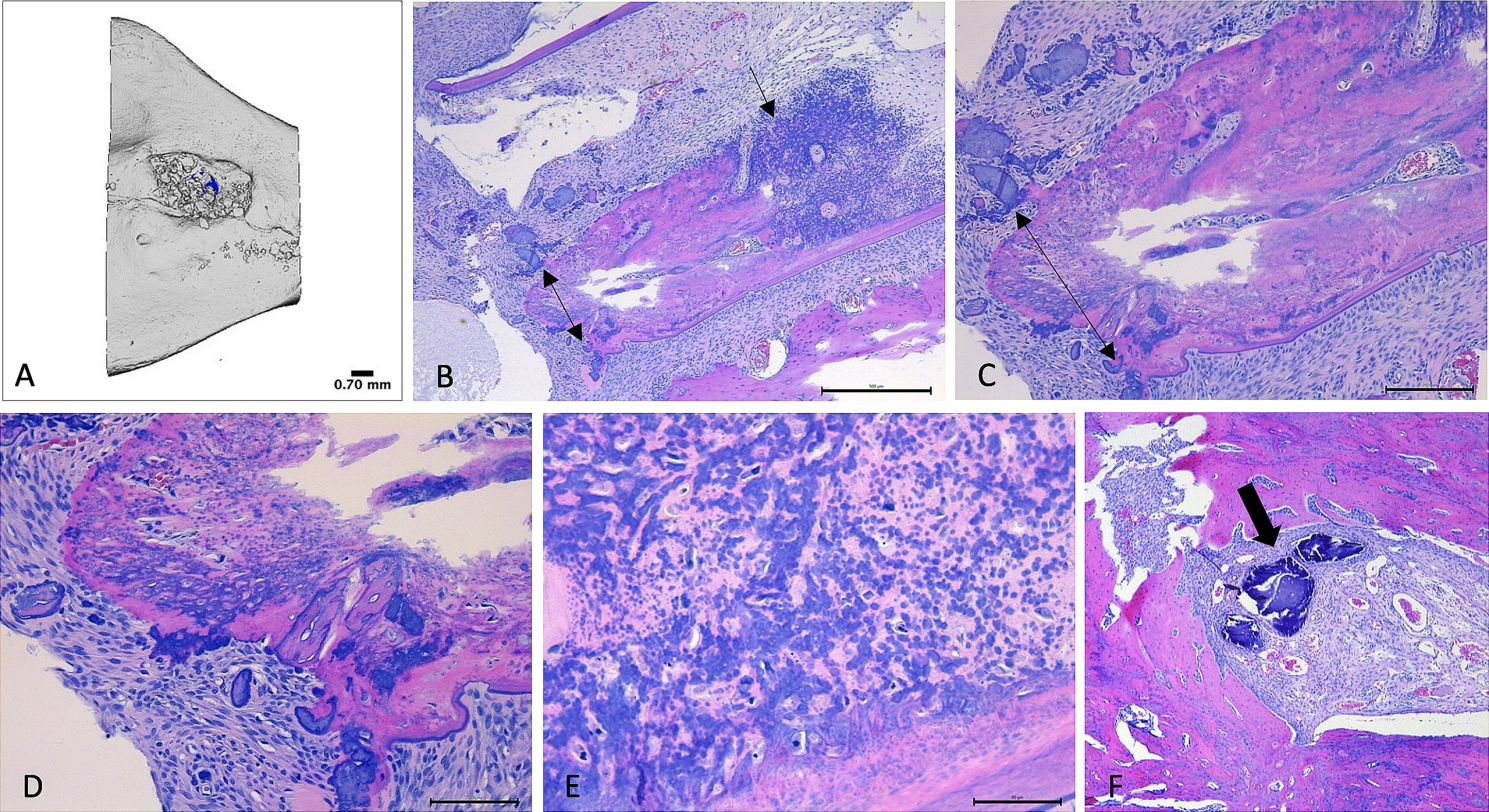
Micro-CT and histopathology images. **(A–E)**: Images **(A)** – Micro-CT, **(B–E)** Histology) collected from right hemimandible that tested positive for SA (Group III; rat 14). **(A)**: Micro-CT reconstructed image of defect area within mandible. Bone particles within irregularly shaped defect indicate presence of Bio-Oss^®^ Collagen. **(B)**: H&E-stained section of hemimandible, demonstrating mandibular bone (pink tissue) with severe, infiltrative inflammation (black arrow). Double-headed arrow highlights areas of necrotic bone. Findings are supportive of osteomyelitis. 5x magnification. **(C)**: 10x magnification of identical bone section. Double-headed arrow highlights areas of necrotic bone. **(D)**: 20x magnification of identical bone section. Demonstrates areas of necrotic bone (dark purple), amid bone tissue (pink) that is infiltrated and distorted by inflammatory infiltrates. **(E)**: 40x magnification of identical bone section, demonstrating infiltration and distortion of bone by inflammatory infiltrates amid areas of necrosis (light pink). **(F)**: H&E-stained histology section, 5x magnification, of right hemimandible that was culture negative for SA (Group II, rat 10). Healthy mandibular bone (pink) surrounds a defect area containing Bio-Oss^®^ Collagen (thick arrow = bone particles) that has been infiltrated by blood vessels.

Severe, necrotizing osteomyelitis was detected in specimens from one rat in Group III that was SA culture negative and PCR positive for SA in bone. Osteomyelitis in this sample was characterized by widespread suppurative inflammation, with areas of necrosis, bone destruction and extension of suppurative inflammation into the root of the right mandibular incisor (tooth 401). Osteomyelitis was not detected in the histopathology slides from other samples (**Figure 7**).

## 5 Discussion

Investigating the pathomechanisms and pathogenic capabilities of SA sequence types as they emerge and cause disease will aid in trend recognition and contribute valuable information to our understanding of disease pathogenesis, and may guide disease intervention. Despite originating in livestock such as cattle and sheep, ST398 has now been recognized as an important human pathogen (Smith and Pearson, 2011). ST398 was isolated by our laboratory from goats with hypertrophic osteomyelitis following orthopedic surgery (Abouelkhair et al., 2018). This finding alone is significant, as there are few reports of ST398 affecting goats (Loncaric et al., 2013; Abouelkhair et al., 2018). In conjunction with the knowledge that SA osteomyelitis is a devastating, progressive disease that persists despite modern therapeutics (Masters et al., 2019; Muthukrishnan et al., 2019), this finding warrants an investigation into sequence type-related disease characteristics.

Our *in vitro* investigation demonstrated the ability of SA to induce apoptosis of MC3T3-E1 cells, stimulate production of IL-6 and reduce production of OPN and OPG in cell culture, with subtle variation between strain types. Specifically, *in vitro* results demonstrated IL-6 production from SA infected cells throughout a 48-hour period paralleled by initial production and subsequent absence of OPN and OPG, regulators of bone destruction and formation, respectively. IL-6 expression peaked between 24 and 48 hours and apoptosis was greater at 20 hours, compared to four hours, supporting the hypothesis that SA induces a pro-inflammatory and apoptotic environment. This confirms that *in vitro* SA infection stimulates apoptosis and inflammation, and suggests that there may be strain dependent differences in virulence of SA causing osteomyelitis. While IL-6 is recognized to be produced throughout the course of osteomyelitis, and has been found to be similarly useful in diagnosis of chronic osteomyelitis as C-reactive protein (CRP) and erythrocyte sedimentation rate (ESR) (Zhao et al., 2021). There remains a question of the specific role of IL-6 in SA infection and clinical osteomyelitis; whether IL-6 production is protective of the affected bone tissue, recruiting the innate immune system to respond to infectious and inflammatory stimuli, or if IL-6 production may contribute to inflammatory stimuli that trigger progressive bone destruction (Marriott et al., 2004). This is relevant, as an important feature of osteomyelitis is alterations in osteoregulation, or the balance between bone formation and destruction (Josse et al., 2015). OPG production has been documented to decrease following SA infection, which suggests a decreased ability to inhibit osteoclastogenesis (Young et al., 2011) which likely leads to increased bone destruction. Our results suggest that an initial production of IL-6 may reflect a protective effect on osteoregulatory mechanisms that then transitions to a dysregulatory effect, evidenced by impaired production of OPG and OPN. It is possible that the release of SA from apoptotic cells triggers an increase in IL-6. Investigation of viability of SA released from apoptotic cells would be valuable, to understand if increases in IL-6 expression are secondary to viable SA cells infecting new osteocytes, or if IL-6 expression increases as a result of non-viable SA release and subsequent immune system response.

*In vitro* characterization demonstrated the ability of SA to invade and persist within immature osteoblasts in cell culture, with differences in cell morphology and number of bacteria present between strain types. USA300 was most capable at achieving intracellular invasion and Cowan1 the least capable within our given timeframe. Cowan1 also exhibited distorted morphology when intracellular, which may indicate a degree of cell damage. A particularly intriguing discovery on TEM imaging was extracellular ST398 and USA300. ST398 was located intracellularly and had numerous organisms located extracellularly along cellular membranes. Those that were located in closer proximity to MC3T3-E1 cells were observed to have halos of material matching the appearance of cell membrane surrounding them. This likely represents a component of the engulfment process, and although a specific mechanism cannot be identified and described solely *via* TEM, suggests that ST398 may have a slightly different or potentially delayed mechanism of intracellular invasion compared to USA300. Based on our selected timeframe, it is unlikely that the cellular membrane-adjacent ST398 were present secondary to initial invasion, apoptosis and subsequent re-localization to a fresh pre-osteoblastic cell. While this may have been plausible if TEM imaging was performed 20 – 24 hours following SA inoculation, our TEM images were collected 90 minutes after inoculation, leaving a delayed mechanism of intracellular invasion as the most reasonable explanation. There were multiple USA300 bacteria that appeared alongside a discontinuous MC3T3-E1 cellular membrane. These findings may represent the previously documented phenomena of extended filipodia to engulf the bacteria (Hudson et al., 1995). Discontinuous cellular membranes may also represent apoptotic cells releasing bacteria, or impairment of cell membrane integrity secondary to fixation and sample processing. Artifact secondary to fixation and processing is considered to be less likely, due to the low frequency with which discontinuous cell membranes were observed.

Intracellular sequestration of SA is recognized as a potential contributor to recurrent or chronic osteomyelitis (Tucker et al., 2000), although specific mechanisms and duration of intracellular persistence are poorly understood. It is possible that intracellular invasion of osteoblastic cells may occur as a mechanism to evade initial immune system detection. If this is the case, intracellular invasion and persistence may be short-lived. It is hypothesized that invaded cells may then be triggered to undergo apoptosis, ultimately contributing to a pro-inflammatory state and dysregulation of bone homeostasis through altered cell signaling (Meghji et al., 1998; Tucker et al., 2000; Josse et al., 2015). It has also been postulated that intracellular invasion is an inefficient method of immune system evasion (Ngo et al., 2022) and is rather a method of SA to establish sequestered bacterial reservoirs. This may contribute to SA’s ability to cause persistent or recurrent osteomyelitis (Tucker et al., 2000; Alexander and Hudson, 2001). There is valuable discussion regarding cell ability to differentiate between viable and non-viable intracellular bacteria (Ngo et al., 2022). This raises questions regarding length of intracellular SA survival. It is possible that SA may persist in a quiescent state within osteocytes until osteocyte death, at which time SA is released and may reinfect other viable bone cells (Ellington et al., 2003). It is likely that at the time of cell death, whether apoptotic or necrotic in nature, SA is released and stimulates an increase in local inflammatory responses. The *in vivo* relevance of intracellular SA in the pathogenesis of osteomyelitis remains to be determined. While *in vitro* evidence is certainly compelling, substantial *in vivo* documentation is lacking. There are unique instances that led to TEM identification of intracellular SA (Bosse at al., 2005), but reports are sparse, which may be a reflection of minimal surveillance.

An *in vivo* challenge was performed to investigate the capability of ST398 strain UTCVM1 as an interspecies bone pathogen. While our *in vitro* work was performed on three SA isolates in tandem, our primary focus within the *in vivo* challenge was an initial investigation into the ability of ST398 strain UTCVM1 to induce osteomyelitis in a rat mandible defect model. A secondary benefit of this model was the ability to investigate initial clearance of ST398 *via* local antimicrobial-laden implant placement. This model, based upon previously published literature (Sodnom-Ish et al., 2021), and fulfilling the main tenants of an animal model of osteomyelitis (Billings and Anderson, 2022), had a success rate of 50% in rats inoculated with SA but not treated with any antibiotic and 20% of rats challenged with SA and a low dose of antibiotic. Success was defined as clinical osteomyelitis lesions and positive SA recovery. Recovered SA had identical antimicrobial susceptibility profiles to the inoculated SA. Affected animals expressed IL-6 and RANKL, a marker of osteoclastogenesis (Boyce and Xing, 2007) over the study period, demonstrating increases in IL-6 expression at weeks two and three, and RANKL expression that increased, followed by a return closer to baseline by week four. Expression of IL-6 and RANKL, although demonstrating no statistically significant differences between experimental groups, again highlight the intersection of inflammation and bone remodeling. These findings align with previous discussions that highlight SA induction of pro-inflammatory states that enhance bone resorption and osteoclastogenesis (Josse et al., 2015). Additionally, while on an extended timeline compared to *in vitro* results, the pattern of a pro-inflammatory state and osteodysregulation is consistent. This is evidenced by the increase in IL-6 both *in vitro* and *in vivo*, as well as impaired OPN and OPG in infected *in vitro* samples, alongside increased RANKL expression *in vivo*.

When observed, histological evidence of osteomyelitis was quite pronounced and confirmed the ability of ST398 strain UTCVM1 to induce inflammation as well as bone remodeling and destruction. The chosen model, while fulfilling the main tenants of animal modeling, i.e. bone trauma, local inoculation of a high dose of planktonic bacteria and placement of foreign material, was inconsistent in establishing clinical, microbiologic, and histological osteomyelitis. This may reflect the need to alter the experimental protocol, either by including a sclerosing agent (Mader, 1985), utilizing a higher bacterial inoculation dose to overcome the impressive ability of the rat’s immune system to clear acute peripheral infections (Reizner et al., 2015), or may accurately reflect an inconsistent ability of ST398 to induce osteomyelitis in the rat.

## 6 Limitations

Histological examination was minimally rewarding, which is thought to be due in part to tissue sectioning. Authors suggest sectioning hemimandibles coronally to separate incisor teeth from caudal bone tissue prior to embedding tissues in paraffin. This may improve sectioning of tissues and provide more accurate slices of bone tissue within the ROI. Recovery of SA from the rats required a combination of culture and PCR and samples from bone and soft tissues. This work was performed utilizing *in vitro* cell culture and an *in vivo* rat model. While this is the appropriate stepwise approach to performing translational research, it is always possible that *in vitro* and animal modeling results will not accurately portray disease behavior in clinical patients.

## 7 Conclusions and future directions

Understanding Staphylococcal strain differences is valuable. SA is a dangerous, versatile human pathogen that may transition from commensal organism (Kluytmans et al., 1997) to disease-causing agent, either within the same individual, between individuals (Bhattacharya et al., 2016), or cross-species (Smith and Pearson, 2011). This work demonstrates the ability of ST398 strain UTCVM1 to establish intracellular infection, drive apoptosis, create a pro-inflammatory state and alter osteoregulatory pathways *in vitro* and *in vivo*. TEM imaging displays intriguing cell membrane activity that may be indicative of cellular internalization mechanisms. In an *in vivo* rat model, ST398 strain UTCVM1 is an inconsistent driver of osteomyelitis, although when successful, is capable of causing clinical, microbiologic and histologic evidence of osteomyelitis. Ultimately, these findings support that ST398 is a competent osteomyelitis pathogen. Given the reports of ST398 as a dangerous, even lethal human pathogen, further investigation of pathogenesis ST398 induced osteomyelitis is warranted. This may include an *in vivo* dose curve of ST398 in comparison to a more widely characterized sequence type, such as USA300 or UAMS1 to add clarity regarding the interspecies potential of ST398 to establish osteomyelitis. It is suggested that this occur either in rats or rabbits, as rabbits are well-suited to dose-curves and pathogenesis investigation (Billings and Anderson, 2022). Once established, there may be value in investigating for intracellular bacteria from harvested bone, whether that is through TEM or *in vivo* monitoring techniques to evaluate viability of bone cells. Additionally, authors suggest to utilize microbiology techniques in conjunction with PCR, to improve sensitivity of SA detection.

## Data availability statement

The raw data supporting the conclusions of this article will be made available by the authors, without undue reservation.

## Ethics statement

The animal study was reviewed and approved by University of Tennessee Institutional Animal Care and Use Committee.

## Author contributions

Conceptualization: CB, RR, MA, SK, and DA. Methodology: RR, AB, MA, SK, CB, SR, and DA. TEM: JK and CB.. Microbiology: RJ, SR, SK, MA, and CB. Writing: CB. Editing: CB, RJ, MA, SK, SR, and DA. Supervision: DA. All authors contributed to the article and approved the submitted version.

## Funding

Funding for *in vitro* experiments was provided by Center of Excellence in Livestock Diseases and Human Health, College of Veterinary Medicine, University of Tennessee.

## Acknowledgments

Dr. John Dunlap for assistance with TEM imaging, Dr. Xiaocun Sun and Dr. Xiaojuan Zhu for statistical support, Elizabeth Croy, Chris Carter and Niloo Khajeh-Kazerooni for technical support, Dr. Sherry Cox for HPLC analysis, Tanya Garcia-Nolen for micro-CT analysis, Dr. Linden Craig for histology consultation, and Dr. Lear for providing BSL-2 laboratory space.

## Conflict of interest

The authors declare that the research was conducted in the absence of any commercial or financial relationships that could be construed as a potential conflict of interest.

## Publisher’s note

All claims expressed in this article are solely those of the authors and do not necessarily represent those of their affiliated organizations, or those of the publisher, the editors and the reviewers. Any product that may be evaluated in this article, or claim that may be made by its manufacturer, is not guaranteed or endorsed by the publisher.
