# Corrigendum: *In vitro* and *In vivo* assessment of caprine origin Staphylococcus aureus ST398 strain UTCVM1 as an osteomyelitis pathogen

**DOI:** 10.3389/fcimb.2022.1112995

**Published:** 2023-01-16

**Authors:** Caroline Billings, Rebecca Rifkin, Mohamed Abouelkhair, Rebekah Duckett Jones, Austin Bow, Jaydeep Kolape, Sreekumari Rajeev, Stephen Kania, David E. Anderson

**Affiliations:** ^1^ Large Animal Clinical Sciences, College of Veterinary Medicine, University of Tennessee, Knoxville, TN, United States; ^2^ Biomedical and Diagnostic Sciences, College of Veterinary Medicine, University of Tennessee, Knoxville, TN, United States; ^3^ Advanced Microscopy and Imaging Center, University of Tennessee, Knoxville, TN, United States

**Keywords:** osteomyelitis, *Staphylococcus aureus*, *in vitro*, *in vivo*, intracellular invasion

## Missing citations

In the published article, certain citations were incorrectly inserted during the typesetting and publication process. Text requiring updated citations is included below.

## 1 Introduction

There is a need to gain deeper understanding of immune system evasion, particularly the significance of intracellular invasion. Recently, it has been reported that the ability of SA to gain intracellular osteoblast access may not only contribute to immune system evasion but may also play a role in shifting osteoblastic activity to induce inflammatory bone pathology (Mendoza Bertelli et al., 2016, Alexander and Hudson, 2001). While SA and methicillin-resistant *S. aureus* (MRSA) are recognized as common etiologic agents of osteomyelitis (Urish and Cassat, 2020), there remains work to be done in identifying specific causative sequence types (ST) and clonal complexes (CC) (Pimental de Araujo et al., 2021). Thorough characterization of the molecular epidemiology of SA involved in osteomyelitis may contribute to understanding of pathogenesis (Post et al., 2014) and strengthen therapeutic strategies, whether those are pre-existing strategies, novel techniques, or combinations thereof.

Along this vein of investigation, SA ST398 has been increasingly recognized as an important human pathogen (Rasigade et al., 2010, Valentin-Domelier et al., 2011, van der Mee-Marquet et al., 2011, Kashif et al., 2019). Previously thought to be primarily a colonizer and occasional pathogen of livestock (de Neeling et al., 2007, Wulf and Voss, 2008), ST398 is now a known causative organism of serious human infections, sometimes carrying methicillin resistance (Wulf and Voss, 2008, Schijffelen et al., 2010, Witte et al., 2007, Armand-Lefevre et al., 2005) and occurring in the absence of contact with livestock (Murra et al., 2019). Human illnesses caused by ST398 include soft tissue infections (Murra et al., 2019), joint infections and osteomyelitis (Post et al., 2014, Park et al., 2019, Senneville et al., 2014), blood-stream infections (BSI) (Valentin-Domelier et al., 2011), and pneumonia, including ventilator-associated pneumonia and lethal, necrotizing pneumonia (Rasigade et al., 2010). Virulence factors and pathogenesis of ST398 are incompletely understood (Smith and Pearson, 2011). Further investigations of CC398 and ST398 in particular are imperative to continue advancing the understanding of Staphylococcal osteomyelitis, with the ultimate goals of enhancing therapeutic strategies, minimizing negative impact to the patient, and reducing overall burden on the healthcare system.

## 2 Materials and methods

### 2.1 *In vitro* methodology

#### 2.1.1 Bacterial strain selection and preparation

Bacterial inoculums were prepared for cell culture by growing each of the three bacterial strain types overnight in 5mL of tryptic soy both (TSB) at 35°C with aeration (Skov et al., 2009, Cunha 2005, Prescott and Klein, 2002). Bacteria were harvested by centrifugation (10 minutes at 4300 X *g*), washed twice in 5mL of Hank’s balanced salt solution (HBSS) and resuspended in Minimum Essential Media α (α-MEM) (Thermo Fisher Scientific) with 10% fetal bovine serum (FBS) as described by Tucker et al. (2000).

#### 2.1.2 Cell culture and bacterial inoculation

Commercially obtained MC3T3-E1 subclone 4 cells (ATCC) were utilized for *in vitro* experiments. MC3T3-E1 cells are immature osteoblastic cells of murine origin. Cells were expanded in tissue culture treated polystyrene flasks and incubated under standard conditions (37°C and 5% CO_2_) in α-MEM media with 10% (FBS), 1% penicillin streptomycin (pen-strep) and amphotericin B. Media was changed every 2 to 3 days. Once cells reached approximately 90% confluency, they underwent enzymatic release from the growth substrate utilizing 0.25% Trypsin-EDTA solution for 2 minutes at 37°C (Jackson et al., 2018). Cells were collected and allocated to experimental set-up.

#### 2.1.5 Osteoregulatory cytokine analysis

Control and experimental samples in tissue-culture treated plates in biologic triplicates were dedicated to cytokine analysis to evaluate bone signaling and remodeling. Samples underwent supernatant collection, washing and media replacement immediately following bacterial inoculation, (time 0). Following this sample collection, media was replaced with osteoinductive media (α-MEM media supplemented with 10mM beta glycerophosphate, 10nM dexamethasone and 155μM ascorbic acid) (Bow et al., 2020) that was changed every 2-3 days. Sampling procedure was repeated at days 7, 14 and 21. Supernatant was saved at -80°C. Concentrations of osteoprotegerin (OPG) and osteopontin (OPN) were determined by ELISA analysis per manufacturer instruction (R&D Systems, Minneapolis, MN. USA). Optical densities of samples and standards were measured at 450nm with an iMark microplate reader (BioRad).

### 2.2 *In vitro* challenge

#### 2.2.1 Rat mandibular defect model

Bacterial osteomyelitis was induced using a procedure adapted from Sodnom-Ish et al. (Sodnom-Ish et al., 2021). Briefly, the procedure operated under the premise of creating bone trauma, introducing a high dose of planktonic bacteria locally, and placing foreign material within traumatized bone to provide a nidus for bacterial colonization. This procedure fulfills the main tenants of osteomyelitis induction in animal models (Billings and Anderson 2022, Guarch-Pérez et al., 2021).

#### 2.2.2 Material description

Bio-Oss^®^ Collagen was utilized as dual drug delivery device and scaffold material to fill space and serve as a potential nidus for bacterial colonization within the defect site. Bio-Oss^®^ is a biocompatible bone mineral matrix composed of 90% purified, bovine-derived small bone particles and 10% porcine collagen (AG GP, 2013). Bio-Oss^®^ Collagen is provided as a sterilized block of varying sizes that can be trimmed into desired size and shape either dry or moistened. There are interconnected pores that extend throughout the material which contribute to hydrophilicity of the device, and allow for simple hydration of the matrix (AG GP, 2013). This material is labeled and utilized for defect-filling and guided bone regeneration (GBR) (AG GP, 2013, Galindo-Moreno et al., 2010).

## 4 Results

### 4.1 *In vitro* characterization

#### 4.1.1 Apoptosis assay

Death induction of bone cells is an important feature of SA osteomyelitis (Josse et al., 2015). We measured apoptosis to better understand the *in vitro* cytotoxicity of selected SA strains. Results (Figure 1) are reported as mean percent of cells undergoing apoptosis when counting 10,000 cell events. USA300 appeared to induce a more severe apoptotic effect (22.1% at 4hr, 80.6% at 20hr) compared to ST398 (30% at 4hr, 64.2% at 20hr). Cells infected with Cowan1 underwent apoptosis to a lesser extent (15% at 4hr, 44.1% at 20hr). Uninfected cells acted as a control (9% at 4hr, 35% at 20hr).

#### 4.1.4 Transmission electron microscopy

Proportions of intracellular and extracellular SA differed between strain types (Figure 3). Additionally, morphologic differences were observed between SA sequence types within infected pre-osteoblastic cells. Cowan1 appeared weakly infective, with few intracellular bacteria and no observed extracellular bacteria. Intracellular Cowan1 displayed distorted shapes. USA300 appeared to be highly infective, with many infected pre-osteoblastic cells. Many infected cells contained more than one bacterial organism. Extracellular USA300 was observed. There was also evidence of USA300 bacterial organisms along interrupted pre-osteoblastic cellular membranes. These likely represent extension of filipodia to incorporate USA300 into the cell (Guarch-Pérez et al., 2021) as well as cell rupture. ST398 bacterial organisms with undisturbed morphology were observed within pre-osteoblastic cells, confirming the ability of ST398 to establish intracellular infection. No infected cell contained more than one bacterial organism. Extracellular ST398 was observed. Many extracellular ST398 were in contact with pre-osteoblastic cellular membrane and were encircled by membranous material.

#### 4.2.2 SA Recovery and characterization

SA was identified in four rats (Group IV, n=3; Group III, n=1) by *in vitro* culture or PCR analysis. Microbiologic cultures yielded positive SA cultures from the bone of two animals from Group IV (rats 18 and 21), along with incidental organisms, including: *Escherichia coli, Staphylococcus xylosus*, and *α-Streptococcus.* PCR analysis demonstrated the presence of SA in the bone of two animals from Group IV (rats 21 and 23) and one from Group III (rat 14), and the soft tissues of two animals from Group IV (rats 21 and 23). This work used the SA MLST database at the University of Oxford (https://pubmlst.org/saureus) (Solyman et al., 2013) for whole genome multilocus sequence typing. Both SA isolates recovered from microbiologic culture were ascribed to ST398 as described previously (Abouelkhair et al., 2018). Ultimately, four (23.5%) out of 17 inoculated rats developed clinical, histologic or microbiologic evidence of SA ST398 osteomyelitis. Of the four SA+ rats, one was in Group III, having been inoculated with SA in the presence of low dose vancomycin (one out of five Group III rats; 20%); three were in Group IV, having been inoculated with SA without antibiotics (three out of six Group IV rats; 50%). The recovered SA had identical antimicrobial susceptibility profiles to the inoculated SA Table 1).

## 5 Discussion

Investigating the pathomechanisms and pathogenic capabilities of SA sequence types as they emerge and cause disease will aid in trend recognition and contribute valuable information to our understanding of disease pathogenesis, and may guide disease intervention. Despite originating in livestock such as cattle and sheep, ST398 has now been recognized as an important human pathogen (Smith and Pearson, 2011). ST398 was isolated by our laboratory from goats with hypertrophic osteomyelitis following orthopedic surgery (Abouelkhair et al., 2018). This finding alone is significant, as there are few reports of ST398 affecting goats (Abouelkhair et al., 2018, Loncaric et al., 2013). In conjunction with the knowledge that SA osteomyelitis is a devastating, progressive disease that persists despite modern therapeutics (Masters et al., 2019, Muthukrishnan et al., 2019), this finding warrants an investigation into sequence type-related disease characteristics.

Our *in vitro* investigation demonstrated the ability of SA to induce apoptosis of MC3T3-E1 cells, stimulate production of IL-6 and reduce production of OPN and OPG in cell culture, with subtle variation between strain types. Specifically, *in vitro* results demonstrated IL-6 production from SA infected cells throughout a 48-hour period paralleled by initial production and subsequent absence of OPN and OPG, regulators of bone destruction and formation, respectively. IL-6 expression peaked between 24 and 48 hours and apoptosis was greater at 20 hours, compared to four hours, supporting the hypothesis that SA induces a pro-inflammatory and apoptotic environment. This confirms that *in vitro* SA infection stimulates apoptosis and inflammation, and suggests that there may be strain dependent differences in virulence of SA causing osteomyelitis. While IL-6 is recognized to be produced throughout the course of osteomyelitis, and has been found to be similarly useful in diagnosis of chronic osteomyelitis as C-reactive protein (CRP) and erythrocyte sedimentation rate (ESR) (Zhao et al., 2021). There remains a question of the specific role of IL-6 in SA infection and clinical osteomyelitis; whether IL-6 production is protective of the affected bone tissue, recruiting the innate immune system to respond to infectious and inflammatory stimuli, or if IL-6 production may contribute to inflammatory stimuli that trigger progressive bone destruction (Marriott et al., 2004). This is relevant, as an important feature of osteomyelitis is alterations in osteoregulation, or the balance between bone formation and destruction (Josse et al., 2015). OPG production has been documented to decrease following SA infection, which suggests a decreased ability to inhibit osteoclastogenesis (Young et al., 2011) which likely leads to increased bone destruction. Our results suggest that an initial production of IL-6 may reflect a protective effect on osteoregulatory mechanisms that then transitions to a dysregulatory effect, evidenced by impaired production of OPG and OPN. It is possible that the release of SA from apoptotic cells triggers an increase in IL-6. Investigation of viability of SA released from apoptotic cells would be valuable, to understand if increases in IL-6 expression are secondary to viable SA cells infecting new osteocytes, or if IL-6 expression increases as a result of non-viable SA release and subsequent immune system response.

*In vitro* characterization demonstrated the ability of SA to invade and persist within immature osteoblasts in cell culture, with differences in cell morphology and number of bacteria present between strain types. USA300 was most capable at achieving intracellular invasion and Cowan1 the least capable within our given timeframe. Cowan1 also exhibited distorted morphology when intracellular, which may indicate a degree of cell damage. A particularly intriguing discovery on TEM imaging was extracellular ST398 and USA300. ST398 was located intracellularly and had numerous organisms located extracellularly along cellular membranes. Those that were located in closer proximity to MC3T3-E1 cells were observed to have halos of material matching the appearance of cell membrane surrounding them. This likely represents a component of the engulfment process, and although a specific mechanism cannot be identified and described solely *via* TEM, suggests that ST398 may have a slightly different or potentially delayed mechanism of intracellular invasion compared to USA300. Based on our selected timeframe, it is unlikely that the cellular membrane-adjacent ST398 were present secondary to initial invasion, apoptosis and subsequent re-localization to a fresh pre-osteoblastic cell. While this may have been plausible if TEM imaging was performed 20 – 24 hours following SA inoculation, our TEM images were collected 90 minutes after inoculation, leaving a delayed mechanism of intracellular invasion as the most reasonable explanation. There were multiple USA300 bacteria that appeared alongside a discontinuous MC3T3-E1 cellular membrane. These findings may represent the previously documented phenomena of extended filipodia to engulf the bacteria (Hudson et al., 1995). Discontinuous cellular membranes may also represent apoptotic cells releasing bacteria, or impairment of cell membrane integrity secondary to fixation and sample processing. Artifact secondary to fixation and processing is considered to be less likely, due to the low frequency with which discontinuous cell membranes were observed.

Intracellular sequestration of SA is recognized as a potential contributor to recurrent or chronic osteomyelitis (Tucker et al., 2000), although specific mechanisms and duration of intracellular persistence are poorly understood. It is possible that intracellular invasion of osteoblastic cells may occur as a mechanism to evade initial immune system detection. If this is the case, intracellular invasion and persistence may be short-lived. It is hypothesized that invaded cells may then be triggered to undergo apoptosis, ultimately contributing to a pro-inflammatory state and dysregulation of bone homeostasis through altered cell signaling (Tucker et al., 2000, Josse et al., 2015, Meghji et al., 1998). It has also been postulated that intracellular invasion is an inefficient method of immune system evasion (Ngo et al., 2022), and is rather a method of SA to establish sequestered bacterial reservoirs. This may contribute to SA’s ability to cause persistent or recurrent osteomyelitis (Tucker et al., 2000, Alexander and Hudson, 2001). There is valuable discussion regarding cell ability to differentiate between viable and non-viable intracellular bacteria (Ngo et al., 2022). This raises questions regarding length of intracellular SA survival. It is possible that SA may persist in a quiescent state within osteocytes until osteocyte death, at which time SA is released and may reinfect other viable bone cells (Ellington et al., 2003). It is likely that at the time of cell death, whether apoptotic or necrotic in nature, SA is released and stimulates an increase in local inflammatory responses. The *in vivo* relevance of intracellular SA in the pathogenesis of osteomyelitis remains to be determined. While *in vitro* evidence is certainly compelling, substantial *in vivo* documentation is lacking. There are unique instances that led to TEM identification of intracellular SA (Bosse et al., 2005), but reports are sparse, which may be a reflection of minimal surveillance.

An *in vivo* challenge was performed to investigate the capability of ST398 strain UTCVM1 as an interspecies bone pathogen. While our *in vitro* work was performed on three SA isolates in tandem, our primary focus within the *in vivo* challenge was an initial investigation into the ability of ST398 strain UTCVM1 to induce osteomyelitis in a rat mandible defect model. A secondary benefit of this model was the ability to investigate initial clearance of ST398 *via* local antimicrobial-laden implant placement. This model, based upon previously published literature (Sodnom-Ish et al., 2021), and fulfilling the main tenants of an animal model of osteomyelitis (Billings and Anderson, 2022), had a success rate of 50% in rats inoculated with SA but not treated with any antibiotic and 20% of rats challenged with SA and a low dose of antibiotic. Success was defined as clinical osteomyelitis lesions and positive SA recovery. Recovered SA had identical antimicrobial susceptibility profiles to the inoculated SA. Affected animals expressed IL-6 and RANKL, a marker of osteoclastogenesis (Boyce and Xing, 2007) over the study period, demonstrating increases in IL-6 expression at weeks two and three, and RANKL expression that increased, followed by a return closer to baseline by week four. Expression of IL-6 and RANKL, although demonstrating no statistically significant differences between experimental groups, again highlight the intersection of inflammation and bone remodeling. These findings align with previous discussions that highlight SA induction of pro-inflammatory states that enhance bone resorption and osteoclastogenesis (Josse et al., 2015). Additionally, while on an extended timeline compared to *in vitro* results, the pattern of a pro-inflammatory state and osteodysregulation is consistent. This is evidenced by the increase in IL-6 both *in vitro* and *in vivo*, as well as impaired OPN and OPG in infected *in vitro* samples, alongside increased RANKL expression *in vivo*.

When observed, histological evidence of osteomyelitis was quite pronounced and confirmed the ability of ST398 strain UTCVM1 to induce inflammation as well as bone remodeling and destruction. The chosen model, while fulfilling the main tenants of animal modeling, i.e. bone trauma, local inoculation of a high dose of planktonic bacteria and placement of foreign material, was inconsistent in establishing clinical, microbiologic, and histological osteomyelitis. This may reflect the need to alter the experimental protocol, either by including a sclerosing agent (Mader 1985), utilizing a higher bacterial inoculation dose to overcome the impressive ability of the rat’s immune system to clear acute peripheral infections (Reizner et al., 2015), or may accurately reflect an inconsistent ability of ST398 to induce osteomyelitis in the rat.

## 6 Limitations

Histological examination was minimally rewarding, which is thought to be due in part to tissue sectioning. Authors suggest sectioning hemimandibles coronally to separate incisor teeth from caudal bone tissue prior to embedding tissues in paraffin. This may improve sectioning of tissues and provide more accurate slices of bone tissue within the ROI. Recovery of SA from the rats required a combination of culture and PCR and samples from bone and soft tissues. This work was performed utilizing *in vitro* cell culture and an *in vivo* rat model. While this is the appropriate stepwise approach to performing translational research, it is always possible that *in vitro* and animal modeling results will not accurately portray disease behavior in clinical patients.

## 7 Conclusions and future directions

Understanding Staphylococcal strain differences is valuable. SA is a dangerous, versatile human pathogen that may transition from commensal organism (Kluytmans et al., 1997) to disease-causing agent, either within the same individual, between individuals (Bhattacharya et al., 2016), or cross-species (Smith and Pearson, 2011). This work demonstrates the ability of ST398 strain UTCVM1 to establish intracellular infection, drive apoptosis, create a pro-inflammatory state and alter osteoregulatory pathways *in vitro* and *in vivo*. TEM imaging displays intriguing cell membrane activity that may be indicative of cellular internalization mechanisms. In an *in vivo* rat model, ST398 strain UTCVM1 is an inconsistent driver of osteomyelitis, although when successful, is capable of causing clinical, microbiologic and histologic evidence of osteomyelitis. Ultimately, these findings support that ST398 is a competent osteomyelitis pathogen. Given the reports of ST398 as a dangerous, even lethal human pathogen, further investigation of pathogenesis ST398 induced osteomyelitis is warranted. This may include an *in vivo* dose curve of ST398 in comparison to a more widely characterized sequence type, such as USA300 or UAMS1 to add clarity regarding the interspecies potential of ST398 to establish osteomyelitis. It is suggested that this occur either in rats or rabbits, as rabbits are well-suited to dose-curves and pathogenesis investigation (Billings and Anderson 2022). Once established, there may be value in investigating for intracellular bacteria from harvested bone, whether that is through TEM or *in vivo* monitoring techniques to evaluate viability of bone cells. Additionally, authors suggest to utilize microbiology techniques in conjunction with PCR, to improve sensitivity of SA detection.

The authors apologize for this error and state that this does not change the scientific conclusions of the article in any way. The original article has been updated.

