# Supplementary figures and images for: In vitro and in vivo assessment of caprine origin Staphylococcus aureus ST398 strain UTCVM1 as an osteomyelitis pathogen
Source: Front Cell Infect Microbiol. 2022 Nov 24;12:1015655. doi: 10.3389/fcimb.2022.1015655 (PMC9885270; doi:10.3389/fcimb.2022.1015655)

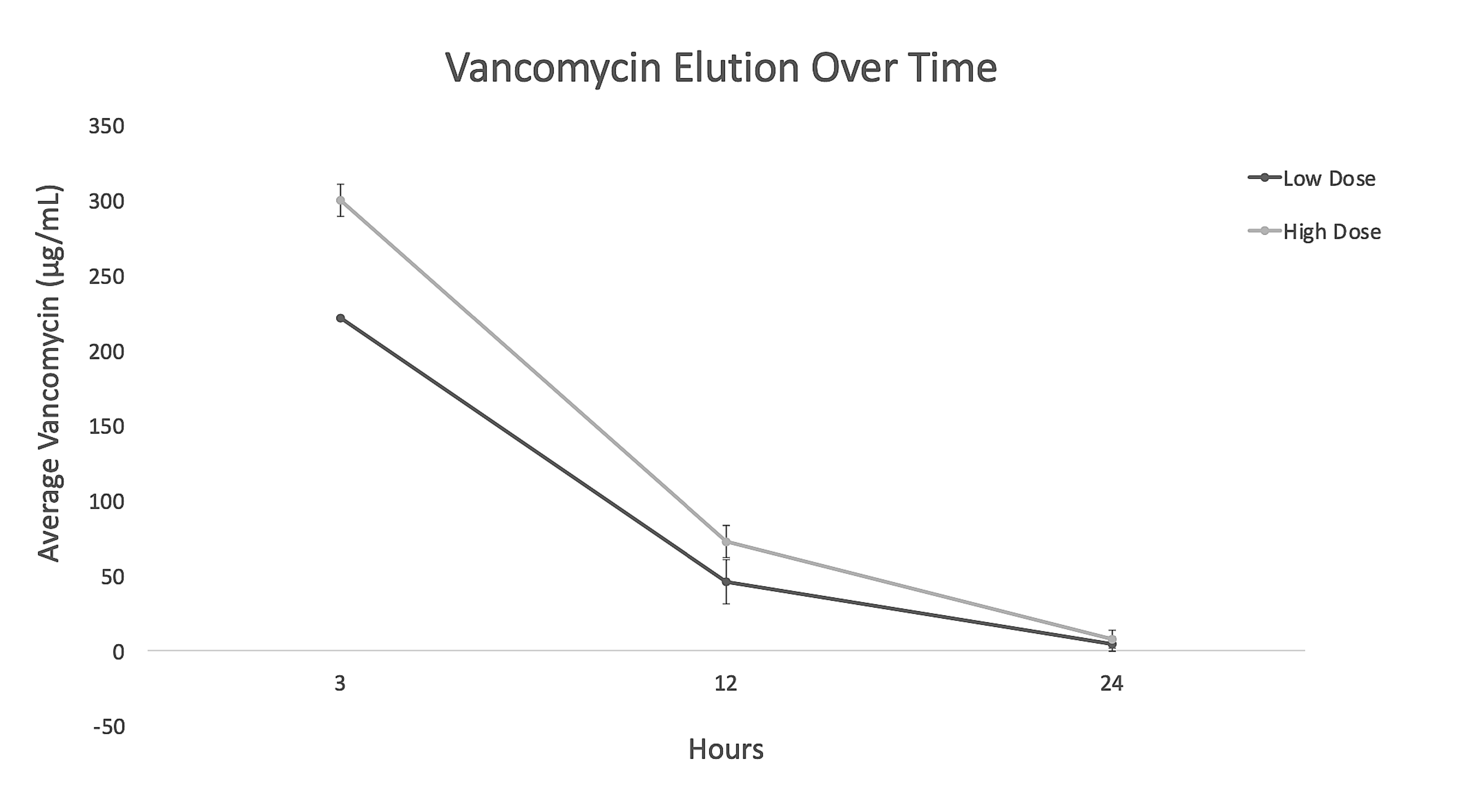

Supplement: Supplementary file 3 [file Image_1.tif]
